# Unveiling the dynamics of emotions in society through an analysis of online social network conversations

**DOI:** 10.1038/s41598-023-41573-9

**Published:** 2023-09-11

**Authors:** Begum Sener, Ezgi Akpinar, M. Berk Ataman

**Affiliations:** 1 McGill University, Montreal, Quebec, Canada; 2 Sabancı University, Istanbul, Turkey; 3 Özyeğin University, Istanbul, Turkey

**Keywords:** Human behaviour, Psychology and behaviour

## Abstract

Social networks can provide insights into the emotions expressed by a society. However, the dynamic nature of emotions presents a significant challenge for policymakers, politicians, and communication professionals who seek to understand and respond to changes in emotions over time. To address this challenge, this paper investigates the frequency, duration, and transition of 24 distinct emotions over a 2-year period, analyzing more than 5 million tweets. The study shows that emotions with lower valence but higher dominance and/or arousal are more prevalent in online social networks. Emotions with higher valence and arousal tend to last longer, while dominant emotions tend to have shorter durations. Emotions occupying the conversations predominantly inhibit others with similar valence and dominance, and higher arousal. Over a month, emotions with similar valences tend to prevail in online social network conversations.

## Introduction

**Figure 1:**
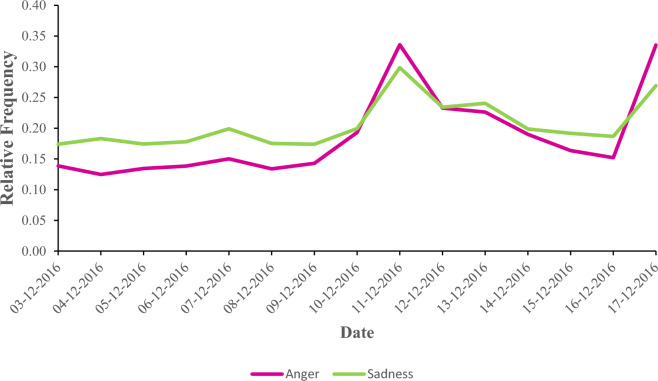
Relative frequencies of anger and sadness expressions on Twitter (in Turkish) in a 15-day time window centered around December 10, 2016 in which a terrorist attack took place. The relative frequencies are calculated as explained in “Materials and methods”. The uptake in anger and sadness frequencies on December 17, 2016, is due to a suspected car bombing.

Millions of messages posted on online social networks, such as Twitter, can be mined to reveal the emotional atmosphere of a society^1^. This atmosphere can be conceived as a palette of emotions, wherein multiple emotions are experienced and expressed simultaneously in the ebb and flow of everyday social life^2^. Temporal exogenous events, major and minor alike (e.g., a terrorist attack, a victory in an international sports championship, untimely passing of a local celebrity, or a humorous incident in a talk show), can make certain emotions prevalent in this palette for a while (e.g., anger, joy, sadness, or amusement). For instance, when the devastating news of a terrorist attack, the stadium bombing in Turkey, hit social networks, conversations of the society on Twitter were occupied with anger more than usual (Fig. 1). On the days that followed, anger continued to occupy the conversations, though increasingly less, alongside the other emotions in the palette expressed at the same or at different rates. In this instance, as anger faded away, it gave way to sadness, which too returned to its normal level after a while. How long should one expect an emotion such as anger to take over the conversations before it returns to its normal level? As anger’s grip loosens, which other emotions can one expect to flourish or wither in the short term, say the next day, or in the long term, say over the course of a month? This paper paints a picture of the dynamic flow of emotions at the aggregate level by describing which emotions are expressed more than others (frequency), how long it takes emotions to return to their steady states after they deviate from them (duration), and which other emotions arise or dwindle as the temporary rise of an emotion(s) ceases (transition) in conversations in online social networks (OSNs).

Understanding the dynamic flow of emotions is crucial, as these aggregate level emotional states are informative for societal outcomes, such as stock market fluctuations^3^, tracking of epidemic diseases^4^, or affective well-being of and life-satisfaction a society^5^. The temporal unfolding of emotions has been studied in the literature, although at an individual level (e.g., Refs.^6,7^). Yet, research on the dynamic flow of a large and diverse set of emotions at an aggregate level is scant, notwithstanding a few exceptions. For instance, using textual analysis of public Twitter messages, it has been shown that there is a temporal pattern of emotions^8,9^ and these patterns may change based on sleep and circadian rhythms^10^ or exogenous events such as COVID-19 outbreaks^11^. These studies mainly show the mere presence of temporal patterns in emotions over time.

We contribute to this burgeoning body of work by exploring the evolution of daily time series of a fine-grained palette of twenty-four discrete emotions, selected from^12^ and ^13^ (Supplementary Table S1), expressed by the members of a society. We obtain these emotion time series by aggregating over a large corpus of tweets (N = 5,068,236), which were sampled randomly from all tweets originated from Turkey-based IPs every day for 790 days and tagged automatically using purpose-built emotion dictionaries. We unpack the dynamic flow of emotions in OSN conversations by estimating a multivariate time series model: a Vector Autoregression (VAR)^14^. VAR models are uniquely suited to studying the temporal dynamics of emotions because they account for both the history of a focal emotion as well as the history of other emotions when predicting the focal emotion's current expression frequency. Moreover, the parameters of a VAR model have a one-to-one correspondence with the components of the dynamic flow we are interested in: the emotions’ average expression frequencies, the duration of emotions, and the transition patterns among emotions.

## Research background and the present study

Existing studies on emotions in OSN conversations mainly focus on the differences in expression frequencies across a handful of emotions (e.g., Refs.^3,15^). Further, research investigates the variation in the likelihood of socially sharing emotions on different platforms^16–18^. These studies explore expression frequency differences at a given point in time, rather than analyzing the temporal shifts. In addition to the differences in the average expression rates of emotions in OSN conversations, we explore how these frequencies evolve over time.

Extant work on the dynamics of emotions has advanced our understanding of the duration of emotions, but at the individual level^19,20^ rather than at the aggregate level. Evidence from behavioral experiments or analysis of experience sampling data demonstrate that some emotions, such as sadness and joy, last longer than others, such as disgust and shame^7,21^. Differences in the duration of emotions felt by individuals have been attributed to appraisals and emotion regulation^22^. Yet, little is known about the persistence of emotions expressed in OSN conversations. We shed light on how long a society is likely to express a specific emotion following a temporal boost before returning to its average frequency level.

Finally, although emotion transitions have been studied to some extent at the aggregate level^23^, majority of the knowledge is based on individual level data. These studies suggest that emotions flow into one another with a pattern^24^. It has been even shown that humans have mental models that can predict the transition of emotions, especially those with similar valence^6^. Research focusing on emotion transitions in the long run finds that, after expressing negative emotions due to a collective trauma, conversations of societies contain expressions of solidarity^23^. In this paper, we reveal short- and long-term transition patterns of emotions expressed in OSN conversations at an aggregate level. In doing so, we show which other emotions the society expresses more—or less—following a temporal boost in one of the emotions, over timeframes ranging from a day to a month.

The dynamic flow of emotions is shaped by a combination of individual and collective mechanisms, although disentangling these processes is not the goal of this paper. Individual level mechanisms, such as emotion contagion^25,26^ and virality^27^, as well as collective level mechanisms, such as emotional sharing feedback loops^23,28,29^ and vicarious emotion learning patterns^30,31^, influence the frequency of emotions, the longevities of these emotions, and the transitions among them. The findings in the literature suggest that the influence of these mechanisms may be boosted or dampened by the underlying appraisal dimensions of valence and arousal. For instance, positive emotions are more contagious than negative ones^32,33^, and last longer^34^. High arousal emotions are more viral than low arousal emotions^35^. Same-valence emotions are shown to follow each other in shorter time periods^32^, and this finding may or may not hold in the long term^23,36^.

Accordingly, we describe the dynamic flow of emotions at the aggregate level by mapping them across their underlying appraisal dimensions. However, different from extant research, we adopt a broader set to better represent the complex world of emotions^37^. Our investigation covers nine interrelated dimensions: valence, arousal, dominance, motivational state, time orientation, agency, un/certainty, attentional activity, and effort^38–41^.

While conducting this study, we take a positivist approach, treating emotions as observable, definable, and quantifiable phenomena as defined by^42^. However, we also acknowledge the prevalent contextual view asserting emotions as cultural and social constructs, not just biological responses^43^. Specifically, we consider emotions as artifacts of cultural contexts, socially constructed and collectively enacted^44^. Recognizing that the experience of emotions is embedded in a cultural context, we create bespoke dictionaries for automated text analysis through native speakers and coders and collect survey data on the underlying appraisal dimensions of emotions from the same cultural milieu. Finally, we acknowledge that tweets are not mere representation of momentary information conduits but social actions^45^ that collectively shape emotional narratives and impact individual responses over time. Therefore, we adopt a modeling approach that accounts the for the historical emotional context when predicting current emotional expression.

## Materials and methods

### Twitter dataset

We obtained data on a random sample of posts on the microblogging site Twitter. The data was provided by IPSOS and retrieved by them using Gnip Inc. (a social media API aggregation company now owned by Twitter) by taking a 1% sample of posts from Turkey-based IPs. The dataset includes 5,068,236 public tweets that were recorded from February 1, 2016, to March 31, 2018 (790 days). For each tweet, we have information on the following: a unique tweet identifier, the date and time of the post, the text of the tweet, and several other variables. The number of tweets per day ranges between 3669 and 13,312 with an average of 6415 tweets (S.D. = 669 tweets).

### Calculating daily emotion frequencies

We obtain the time series needed to model the dynamic flow of emotions in OSN conversations in two steps: labeling and aggregating the tweets. We adopted a lexicon-based approach for labeling the tweets. To that end, we created custom-built emotion dictionaries composed of lemmatized unigrams and bigrams (an n-gram is a continuous sequence of n words in a text), as lexica for the 24 emotions used in this study did not exist. The dictionary formation process consists of four steps. First, we formed seed dictionaries for each emotion based on the words used in their definitions and their synonyms. We then extended these seed dictionaries by adding related words and words from externally validated lexica. Third, we further extended the dictionaries by adding lemmatized unigrams and bigrams from a human-coded sub-sample of tweets. Lastly, because Turkish is an agglutinative language, we populated these dictionaries by adding the derivations of the words’ stems.

Using these dictionaries, we labeled each tweet as containing none, one, or more of the 24 emotions using a matching method (i.e., obtaining a vector of indicator variables showing which emotions are expressed in each tweet). We then aggregated over the tweets, per emotion and per day, to obtain the daily emotion time series. The performance of the custom-built emotion dictionaries was assessed by studying the behavior of the time series around dates of national importance and comparing our time series with the time series obtained by using an externally validated dictionary, NRC^46^, on a set of overlapping emotions. See Supplementary Methods for detailed descriptions of dictionary formation, dictionary validation, automated text analysis, and daily time series construction processes.

### Identifying and measuring underlying emotion dimensions

As our goal is to link the components of the dynamic flow of emotions, such as carryover and transitions, to underlying appraisal dimensions, we conducted a survey to determine the dimension scores of the 24 emotions. To better represent the complex world of emotions, we identified an exhaustive set of dimensions, which includes dominance, motivational state, agency, certainty, attentional activity, effort, and time orientation as well as the frequently used dimensions of valence and arousal. Supplementary Table S3 contains the definitions of the nine appraisal dimensions in the study and Supplementary Survey details the survey conducted to determine the dimension scores of each emotion.

### Analysis approach

We use a multivariate time series model based on a traditional VAR specification to investigate the temporal dynamics of emotions and how its components vary according to the underlying dimensions of emotions (see, for instance^47^ for an alternative application of the general family of VAR models in the analyses of emotion dynamics). The VAR model simultaneously accommodates the following behaviors: (1) carryover in emotions, through the inclusion of the lagged values of emotions’ own frequencies, (2) the transitions to all destination emotions from all other emotions, through the inclusion of lagged values of other emotions’ frequencies, and (3) the co-occurrence of emotions, through a full error variance–covariance matrix. Given our goal of describing this process over a multidimensional appraisal space, we tailor the specification of a classic VAR to fit our need.

Specifically, (1) to understand which emotions are expressed more than others, we link the model parameters capturing an emotion’s average expression frequency to the emotions’ underlying appraisal dimension scores; (2) to shed light on how long a society is likely to express a specific emotion following a temporal boost, we relate the carryover coefficients in the model governing the duration of emotions to the emotions’ underlying appraisal dimension scores; (3) to reveal the transition patterns among emotions in the short term, we express the coefficients capturing the short-term transitions from one emotion to another as a function of the distance between the emotion pairs in the multidimensional appraisal space. We allow the short-term transitions to be asymmetric. That is, the impact of an exogenous increase in an emotion that scores high on one dimension on an emotion that scores low on the same dimension is allowed to be different than that in the other direction, yet proportional to the distance between the emotions (e.g., the impact of a temporary boost in anger on sadness is different from that of sadness on anger). The resultant specification is parsimonious and one that produces interpretable coefficients (see “Model specification”).

Finally, to quantify the long-term transitions among emotions, we use post-estimation impulse-response function (IRF) analyses^48^, piecing together the carryover in, the short-term transitions among, and the co-occurrence of emotions.

### Model specification

The basic form of our VAR model is as follows:

1 $$\left[\begin{array}{c}{\mathrm{E}}_{1,\mathrm{t}}\\ {\mathrm{E}}_{2,\mathrm{t}}\\ \vdots \\ {\mathrm{E}}_{23,\mathrm{t}}\\ {\mathrm{E}}_{24,\mathrm{t}}\end{array}\right]= \left[\begin{array}{c}{\mathrm{\alpha }}_{1}\\ {\mathrm{\alpha }}_{2}\\ \vdots \\ {\mathrm{\alpha }}_{23}\\ {\mathrm{\alpha }}_{24}\end{array}\right]+\left[\begin{array}{ccccc}{\upgamma }_{\mathrm{1,1}}& {\upgamma }_{\mathrm{1,2}}& \cdots & {\upgamma }_{\mathrm{1,23}}& {\upgamma }_{\mathrm{1,24}}\\ {\upgamma }_{\mathrm{2,1}}& {\upgamma }_{\mathrm{2,2}}& \cdots & {\upgamma }_{\mathrm{2,23}}& {\upgamma }_{\mathrm{2,24}}\\ \vdots & \vdots & \ddots & \vdots & \vdots \\ {\upgamma }_{\mathrm{23,1}}& {\upgamma }_{\mathrm{23,2}}& \cdots & {\upgamma }_{\mathrm{23,23}}& {\upgamma }_{\mathrm{23,24}}\\ {\upgamma }_{\mathrm{24,1}}& {\upgamma }_{\mathrm{24,2}}& \cdots & {\upgamma }_{\mathrm{24,23}}& {\upgamma }_{\mathrm{24,24}}\end{array}\right]\left[\begin{array}{c}{\mathrm{E}}_{1,\mathrm{t}-1}\\ {\mathrm{E}}_{2,\mathrm{t}-1}\\ \vdots \\ {\mathrm{E}}_{23,\mathrm{t}-1}\\ {\mathrm{E}}_{24,\mathrm{t}-1}\end{array}\right]+\left[\begin{array}{cc}\begin{array}{c}{\upbeta }_{\mathrm{1,1}}\\ {\upbeta }_{\mathrm{2,1}}\end{array}& \begin{array}{c}{\upbeta }_{\mathrm{1,2}}\\ {\upbeta }_{\mathrm{2,2}}\end{array}\\ \vdots & \vdots \\ \begin{array}{c}{\upbeta }_{\mathrm{23,1}}\\ {\upbeta }_{\mathrm{24,1}}\end{array}& \begin{array}{c}{\upbeta }_{\mathrm{23,2}}\\ {\upbeta }_{\mathrm{24,2}}\end{array}\end{array}\right]\left[\begin{array}{c}{\mathrm{X}}_{1,\mathrm{t}}\\ {\mathrm{X}}_{2,\mathrm{t}}\end{array}\right]+\left[\begin{array}{c}{\upvarepsilon }_{1,\mathrm{t}}\\ {\upvarepsilon }_{2,\mathrm{t}}\\ \vdots \\ {\upvarepsilon }_{23,\mathrm{t}}\\ {\upvarepsilon }_{24,\mathrm{t}}\end{array}\right]$$

The model in Eq. (1) is a VARX model of order one, which we will use to illustrate how we tailor the specification. Extension to higher-order VARX models is straightforward.

E_it_ in Eq. (1) is the relative frequency of emotion i (i = 1,…,24) on day t (t = 1,…,790). X_1t_ and X_2t_ are two exogenous variables included to control for the effects of the 280-characters-long tweet experiment and rollout. These step dummies take the value of 1 after September 26, 2017, and November 7, 2017, respectively. ε_it_ are the white-noise disturbances distributed N(0,∑), where ∑ is a full variance–covariance matrix. α_i_, γ_ij_, and β_i._ are the parameters to be estimated. α_i_ is the base expression level of emotion i. The vector autoregressive parameter matrix contains the carryover coefficients governing the duration of an emotional state (γ_ij_, for all i = j) and the coefficients capturing short-term transitions between emotional states (γ_ij_, for all i ≠ j).

As our goal is to describe whether and to what extent emotion dynamics are associated with the underlying dimensions, we express α_i_ and γ_ij_ as functions of these dimensions. Specifically,

2 $${\upalpha }_{{\text{i}}} = {\upmu }_{0} + \mathop \sum \limits_{{{\text{k}} = 1}}^{{\text{K}}} {\upmu }_{{\text{k}}} \overline{{{\text{DIM}}}}_{{{\text{ik}}}} + {\upmu }_{{\text{S}}} {\overline{\text{S}}}_{{\text{i}}}$$

where DIM_ik_ is emotion i’s score on dimension k (k = 1,…,K). While this section presents the general model specification, exploratory factor analysis of survey data measuring the underlying dimensions reveals that a 3-dimensional space effectively distinguishes between emotions (see “Emotion dimension survey results”). Thus, K = 3 in our empirical analysis. The overbar indicates that the dimension scores are mean centered across emotions. Consequently, μ_0_ captures the base expression level of an emotion scoring average on all dimensions, and μ_k_ captures how base expression level varies along dimension k. As a control, we add dictionary size (S_i_, the total number of unigrams and bigrams used while labeling whether emotion i is present in any given tweet). Similarly, we express the carryover coefficients as a function of the underlying dimensions and control for the effect of dictionary size.

3 $${\upgamma }_{{{\text{ij}}_{{\forall {\text{i}} = {\text{j}}}} }} = {\uplambda }_{0} + \mathop \sum \limits_{{{\text{k}} = 1}}^{{\text{K}}} {\uplambda }_{{\text{k}}} \overline{{{\text{DIM}}}}_{{{\text{ik}}}} + {\uplambda }_{{\text{S}}} {\overline{\text{S}}}_{{\text{i}}}$$

where λ_0_ captures the carryover of an emotion scoring average on all dimensions, and λ_k_ captures how carryover varies along dimension k. Finally, we express γ_ij_ (for all i ≠ j) capturing the short-term transitions from emotion j (i.e., the source emotion) to emotion i (i.e., the destination emotion) as a function of the distance between emotions i and j in the multidimensional appraisal space.

4 $${\upgamma }_{{{\text{ij}}}} = {\uptau }_{0} + \mathop \sum \limits_{{{\text{k}} = 1}}^{{\text{K}}} {\uptau }_{{\text{k}}}^{ + } {\text{DIST}}_{{{\text{ij}}}}^{{{\text{k}} + }} + \mathop \sum \limits_{{{\text{k}} = 1}}^{{\text{K}}} {\uptau }_{{\text{k}}}^{ - } {\text{DIST}}_{{{\text{ij}}}}^{{{\text{k}} - }} + {\uptau }_{{\text{S}}} {\overline{\text{S}}}_{{\text{i}}}$$

where

5a $${\mathrm{DIST}}_{\mathrm{ij}}^{\mathrm{k}+}= \left\{\begin{array}{cc}{\mathrm{DIM}}_{\mathrm{ik}}-{\mathrm{DIM}}_{\mathrm{jk}},& \forall {\mathrm{DIM}}_{\mathrm{ik}}>{\mathrm{DIM}}_{\mathrm{jk}}\\ 0,& \mathrm{otherwise}\end{array}\right\}$$

5b $${\mathrm{DIST}}_{\mathrm{ij}}^{\mathrm{k}-}=\left\{\begin{array}{cc}{\mathrm{DIM}}_{\mathrm{jk}}-{\mathrm{DIM}}_{\mathrm{ik}},& \forall {\mathrm{DIM}}_{\mathrm{jk}}>{\mathrm{DIM}}_{\mathrm{ik}}\\ 0,& \mathrm{otherwise}\end{array}\right\}$$

The distance operationalization in Eqs. (5a) and (5b) allows for directionally asymmetric transitions in Eq. (4). That is, the specification allows the transition from a source emotion scoring low on dimension k to a destination emotion scoring high on the same dimension (i.e., moving up on dimension k) to be different than the transition in the other direction (i.e., moving down on dimension k, with a source emotion scoring higher than the destination emotion).

The meanings of the coefficients in Eq. (4) are different than those in Eq. (2) or Eq. (3). ${\uptau }_{0}$ captures the short-term effect (i.e., next-day effect) of an increase in an emotion on another emotion located exactly on the same spot in the multidimensional appraisal space but labelled differently. Such an emotion exists only theoretically. As such, ${\uptau }_{0}$ alone is not useful and has to be considered together with the other coefficients. ${\uptau }_{\mathrm{k}}^{+}$ captures how the short-term effect changes as the destination emotion moves further away from the source emotion which scores lower on dimension k (i.e., short-term transition from an emotion scoring low on dimension k to a high-scoring emotion). ${\uptau }_{\mathrm{k}}^{-}$ captures how the short-term effect changes as the destination emotion moves further away from the source emotion which scores higher on dimension k (i.e., short-term transition from an emotion scoring high on dimension k to a low-scoring emotion). Plugging in Eqs. (2), (3), and (4) in Eq. (1) and rearranging the terms give the final specification (see Supplementary Model), which we estimate using Feasible Generalized Least Squares.

Finally, in addition to the vector autoregressive matrix coefficient estimates, our post-estimation IRF analyses require, as inputs, exogenous shock vectors that accommodate the instantaneous effects of different emotions on the remaining ones to allow for co-occurrence of emotions. These instantaneous effects are captured in the full error variance–covariance matrix and, assuming multivariate normality, we obtain them by calculating conditional normal expectations—an idea that can be traced to^49^. Specifically, a shock of δ units to emotion i is expected to impact all other j emotions by $\updelta \times {\upsigma }_{\mathrm{ij}}/{\upsigma }_{\mathrm{ii}}$ on the same day, where ${\upsigma }_{\mathrm{ij}}$ is the corresponding element of the error variance–covariance matrix.

## Results

### Emotion dimension survey results

Using the data obtained from the survey measuring the 24 fine-grained emotions over nine interrelated dimensions, we conducted an exploratory factor analysis. The analysis uncovered three higher-order factors, resembling the structure in more recent dimensional spaces^37^. The three factors that emerge closely resemble the classical valence, arousal, and dominance (VAD) framework commonly used in literature^50,51^ but are broader in nature.

The first dimension is composed of valence, time orientation, motivational state, attentional activity, and effort. We label this dimension “valence” (V) for ease of exposition but acknowledge its broader meaning: An emotion scoring high (low) on valence refers to an emotional state that is positive/pleasant (negative/unpleasant), future-oriented (past-oriented), not demanding (demanding) effort but deserving (not deserving) attention, and one wherein the individuals are motivated to approach (avoid). “Arousal” (A) is a stand-alone dimension. Corresponding to its conventional definition, it distinguishes between emotions based on the level of activation: an emotion scoring high (low) on arousal indicates heightened (reduced) levels of activation. Finally, the third dimension, which we label as “dominance” (D) for ease of exposition, is composed of dominance, certainty, and agency. An emotion that scores high (or low) on dominance is one in which the person is certain (or uncertain) about how they feel and claims (or doesn't claim) responsibility and feels (or doesn't feel) independent. Supplementary Table S2 displays the emotions’ scores on these three higher-order dimensions.

### VAR model results

Before estimating the proposed VAR model, whose parameters are expressed as functions of the emotions’ dimension scores, we first checked for stationarity of the emotion time series using Augmented Dickey–Fuller tests and found that all our time series are stationary. Second, we determined the order of the VAR model. Comparison of model fit criteria across VAR models with different lag lengths suggested that the optimal lag length in our case is 1 (see Supplementary Table S6). Third, Granger Causality tests justified the estimation of a dynamic system capable of accounting for contemporaneous and short-term associations among emotions as well as the carryover of emotions (i.e., a VAR model) to unveil the emotion dynamics observed in OSN conversations (see Supplementary Granger Causality Test). Next, we present the results of the proposed VAR model of order one.

#### Frequency

First, we concentrate on the average frequency of an emotion expressed in OSN conversations (i.e., base level). We discover that the base level of an emotion’s frequency decreases with valence (${\upmu }_{\mathrm{V}}$ = − 0.000887, ${\mathrm{SE}}_{{\upmu }_{\mathrm{V}}}$ = 0.000389, ${\mathrm{P}}_{{\upmu }_{\mathrm{V}}}$ = 0.022), and increases with dominance (${\upmu }_{\mathrm{D}}$ = 0.005441, ${\mathrm{SE}}_{{\upmu }_{\mathrm{D}}}$ = 0.000945, ${\mathrm{P}}_{{\upmu }_{\mathrm{D}}}$ < 0.001) and arousal, albeit with a marginal effect (${\upmu }_{\mathrm{A}}$ = 0.000918, ${\mathrm{SE}}_{{\upmu }_{\mathrm{A}}}$ = 0.000499, ${\mathrm{P}}_{{\upmu }_{\mathrm{A}}}$ = 0.065). The emotional palette of this OSN, on average during the 790 days, is composed mainly of emotions that score lower on valence but higher on dominance and arousal.

#### Duration

Governed by the carryover in the time series, duration captures how long it will take an emotion to return to its normal level after being subjected to an exogenous shock. We express the durations of emotions using the P% duration interval. The interval is the number of time periods that passes before P% of the expected effect of an exogenous shock takes place and is given by $\mathrm{ln}(1-\mathrm{P}/100)/\mathrm{ln}(\uplambda ) -1$, where λ is the carryover coefficient^52^. The carryover of an emotion with average valence, arousal, and dominance implies a 90% duration interval of 13.8 days (${\uplambda }_{0}$ = 0.855983, ${\mathrm{SE}}_{{\uplambda }_{0}}$ = 0.003708, ${\mathrm{P}}_{{\uplambda }_{0}}$ < 0.001). That is, holding everything else constant, it takes approximately 13.8 days for 90% of the cumulative effect of an exogenous shock to the emotion time series to materialize.

In terms of the relationship between underlying dimensions and the duration of emotions, we find that the carryover increases with valence (${\uplambda }_{\mathrm{V}}$ = 0.056753, ${\mathrm{SE}}_{{\uplambda }_{\mathrm{V}}}$ = 0.002399, ${\mathrm{P}}_{{\uplambda }_{\mathrm{V}}}$ < 0.001) and arousal (${\uplambda }_{\mathrm{A}}$ = 0.011204, ${\mathrm{SE}}_{{\uplambda }_{\mathrm{A}}}$ = 0.001966, ${\mathrm{P}}_{{\uplambda }_{\mathrm{A}}}$ < 0.001) but decreases with dominance (${\uplambda }_{\mathrm{D}}$ = − 0.165068, ${\mathrm{SE}}_{{\uplambda }_{\mathrm{D}}}$ = 0.006454, ${\mathrm{P}}_{{\uplambda }_{\mathrm{D}}}$ < 0.001). The effect of an exogenous shock to a high-scoring emotion (+ 1 SD) on valence fades slower than that of a low-scoring emotion (− 1 SD)—the 90% duration intervals are 39.3 and 7.7 days, respectively. Similarly, if an emotion with a high (low) arousal score experiences an exogenous shock to its base frequency level, the conversations will be occupied by that emotion for a longer (shorter) period: for high and low arousal emotions, the 90% duration interval is 15.4 and 12.4 days, respectively. Finally, conversations featuring emotions with higher levels of dominance die out faster (90% duration interval is 7.0 days) than those with lower levels (90% duration interval is 59.1 days). In sum, emotions with high valence and/or arousal are more likely to occupy the OSN conversation for longer, whereas emotions with high dominance are less likely to do so.

#### Short-term transition

The short-term transitions are myopic cross-effects governed by cross-lagged associations among the emotion time series (i.e., the change in the frequency level of an emotion on the day after another emotion experiences a change). We find that, when the frequency of an emotion increases on a given day, the frequencies of other emotions positioned extremely close to the source emotion (i.e., to the point that distance becomes negligible) decrease on the following day (${\uptau }_{0}$ = − 0.011279, ${\mathrm{SE}}_{{\uptau }_{0}}$ = 0.000929, ${\mathrm{P}}_{{\uptau }_{0}}$ < 0.001). The magnitude of suppression decreases as the valence distance between the source emotion (i.e., the emotion that experiences the boost) and the destination emotion (i.e., the emotion whose next-day frequency is going to be impacted) increases both in the upward direction (${\uptau }_{\mathrm{V}}^{+}$ = 0.001480, ${\mathrm{SE}}_{{\uptau }_{\mathrm{V}}^{+}}$ = 0.000342, ${\mathrm{P}}_{{\uptau }_{\mathrm{V}}^{+}}$ < 0.001) and in the downward direction $({\uptau }_{\mathrm{V}}^{-}$ = 0.001589, ${\mathrm{SE}}_{{\uptau }_{\mathrm{V}}^{-}}$ = 0.000323, ${\mathrm{P}}_{{\uptau }_{\mathrm{V}}^{-}}$ < 0.001). Valence-distance effect turns out to be symmetric: the source emotion impacts equidistant destination emotions above and below itself identically (χ^2^(1) = 0.04, P = 0.841). The same pattern is observed for dominance: the source emotion suppresses distant destination emotions on the dominance dimension less (${\uptau }_{\mathrm{D}}^{+}$ = 0.003724, ${\mathrm{SE}}_{{\uptau }_{\mathrm{D}}^{+}}$ = 0.000730, ${\mathrm{P}}_{{\uptau }_{\mathrm{D}}^{+}}$ < 0.001 and ${\uptau }_{\mathrm{D}}^{-}$ = 0.0036949, ${\mathrm{SE}}_{{\uptau }_{\mathrm{D}}^{-}}$ = 0.000684, ${\mathrm{P}}_{{\uptau }_{\mathrm{D}}^{-}}$ < 0.001) and in similar magnitudes (χ^2^(1) ≈ 0, P = 0.983). In sum, a boost in a source emotion draws disproportionately more (less) from the neighboring (remote) emotions on the following day than it does from the remote (neighboring) emotions over the valence and dominance continua.

Short-term transitions are influenced differently and asymmetrically by the relative positions of source and destination emotions on the arousal dimension. Following an increase in a source emotion’s frequency, destination emotions with higher levels of arousal are suppressed and increasingly more based on their arousal distance (${\uptau }_{\mathrm{A}}^{+}$ = − 0.001478, ${\mathrm{SE}}_{{\uptau }_{\mathrm{A}}^{+}}$ = 0.000422, ${\mathrm{P}}_{{\uptau }_{\mathrm{A}}^{+}}$ < 0.001). Destination emotions with lower levels of arousal are suppressed as well, but equally, as the arousal distance effect in this direction is insignificant (${\uptau }_{\mathrm{A}}^{-}$ = − 0.000039, ${\mathrm{SE}}_{{\uptau }_{\mathrm{A}}^{-}}$ = 0.000361, ${\mathrm{P}}_{{\uptau }_{\mathrm{A}}^{-}}$ > 0.05). In sum, a boost in a source emotion draws disproportionately more from higher arousal emotions on the following day, and equally, from all lower arousal emotions.

The short-term suppression of all destination emotions in response to a boost in a source emotion coupled with the significant carryover of the boost in the source emotion may suggest at the first glance that the surface area of this emotional palette is constrained (i.e., the temporary rise in one emotion comes at the expense of others). However, the finding that the amount of suppression in destination emotions is non-uniform and varies along the three dimensions, and that the pattern of variation observed along the valence and dominance dimensions is different from that of the arousal dimension, suggest other mechanisms might be at play.

One such mechanism that comes to mind is the imitation in the emotion contagion literature^53^, which may explain especially the pattern of results observed over the valence and dominance continua. If imitation-driven contagion holds, members of the OSN experiencing emotion-eliciting events that could be appraised similarly—but not identically—as the emotion which recently occupied the conversations can label their experiences with that specific emotion, rather than what actually fits (e.g., expressing happiness due to its recent takeover of OSN conversations, even if joy or amusement would have been more appropriate with the actual emotion-eliciting events). The pattern of results observed over the arousal continuum, on the other hand, seems to be guided by a different mechanism, one of emotion regulation. Emotion regulation strategies, such as suppression and reappraisal, can inform how close or far the most impacted destination emotion will be to the source emotion^54^. Our findings suggest a tendency to down-regulate the arousal level of these aggregate-level emotions, probably due to higher-arousal emotions—emotional states of excitement, activation, and stimulation—being more difficult to maintain than their counterparts. If such mechanisms are at play, then they would be visible in associations among emotions at other isolated time slices; for instance, the same day as the source emotion experiences the boost.

#### Co-occurrence

To test this idea, we turn to IRF analysis and examine how the contemporaneous effects of 10% exogenous shocks to different source emotions on destination emotions (i.e., IRFs on day 0) vary as a function of asymmetric distances between emotion pairs. The regression results (Table S8, panel labeled CE Regression) show that, consistent with a contagion prediction, emotions that are close in valence tend to co-occur more than those that are far apart (${\mathrm{CE}}_{\mathrm{C}}$ = 0.021172, ${\mathrm{SE}}_{{\mathrm{CE}}_{\mathrm{C}}}$ = 0.003541, ${\mathrm{P}}_{{\mathrm{CE}}_{\mathrm{C}}}$ < 0.001; ${\mathrm{CE}}_{\mathrm{V}}^{+}$ = − 0.005217, ${\mathrm{SE}}_{{\mathrm{CE}}_{\mathrm{V}}^{+}}$ = 0.001429, ${\mathrm{P}}_{{\mathrm{CE}}_{\mathrm{V}}^{+}}$ < 0.001; ${\mathrm{CE}}_{\mathrm{V}}^{-}$ = − 0.005680, ${\mathrm{SE}}_{{\mathrm{CE}}_{\mathrm{V}}^{-}}$ = 0.001429, ${\mathrm{P}}_{{\mathrm{CE}}_{\mathrm{V}}^{-}}$ < 0.001). So do the emotions along the dominance dimension (${\mathrm{CE}}_{\mathrm{D}}^{+}$ = − 0.004404, ${\mathrm{SE}}_{{\mathrm{CE}}_{\mathrm{D}}^{+}}$ = 0.003507, ${\mathrm{P}}_{{\mathrm{CE}}_{\mathrm{D}}^{+}}$ > 0.100 and ${\mathrm{CE}}_{\mathrm{D}}^{-}$ = − 0.007994, ${\mathrm{SE}}_{{\mathrm{CE}}_{\mathrm{D}}^{-}}$ = 0.003507, ${\mathrm{P}}_{{\mathrm{CE}}_{\mathrm{D}}^{-}}$ < 0.050), albeit an insignificant yet directionally consistent effect for destination emotions scoring above the source emotion. Finally, co-occurrence along the arousal dimension appears to be independent of the distance between emotion pairs (${\mathrm{CE}}_{\mathrm{A}}^{+}$ = − 0.001917, ${\mathrm{SE}}_{{\mathrm{CE}}_{\mathrm{A}}^{+}}$ = 0.001670, ${\mathrm{P}}_{{\mathrm{CE}}_{\mathrm{A}}^{+}}$ > 0.100 and ${\mathrm{CE}}_{\mathrm{A}}^{-}$ = 0.001308, ${\mathrm{SE}}_{{\mathrm{CE}}_{\mathrm{A}}^{-}}$ = 0.001670, ${\mathrm{P}}_{{\mathrm{CE}}_{\mathrm{A}}^{-}}$ > 0.100). However, the directions of the coefficient estimates suggest that lower-arousal (higher-arousal) emotions may co-occur more (less) with the source emotion that experiences the boost, consistent with the tendency to down-regulate on arousal.

These conceptually consistent yet directionally opposite findings obviate the need to assess the combined effect of emotion co-occurrence and short-term transitions among them, and to bring in the carryover in emotions to complete the picture. The long-term transition results discussed below combine the contemporaneous association in emotion frequencies due to co-occurrence with the carryover in emotions and the myopic cross-effects among them.

#### Long-term transition

To investigate the long-term transition among emotions, we trace the impact of a 10% exogenous shock, applied one at a time, to each and every source emotion on all other emotions over a 30-day period, including the day of the shock (day 0). Long-term transition is operationalized as the cumulative IRF (CIRF) between days 1 and 29. The CIRF represents the total incremental change in the frequency of an emotion and indicates how much more—or less—the emotion is expected to occupy OSN conversations in the long run.

**Figure 2:**
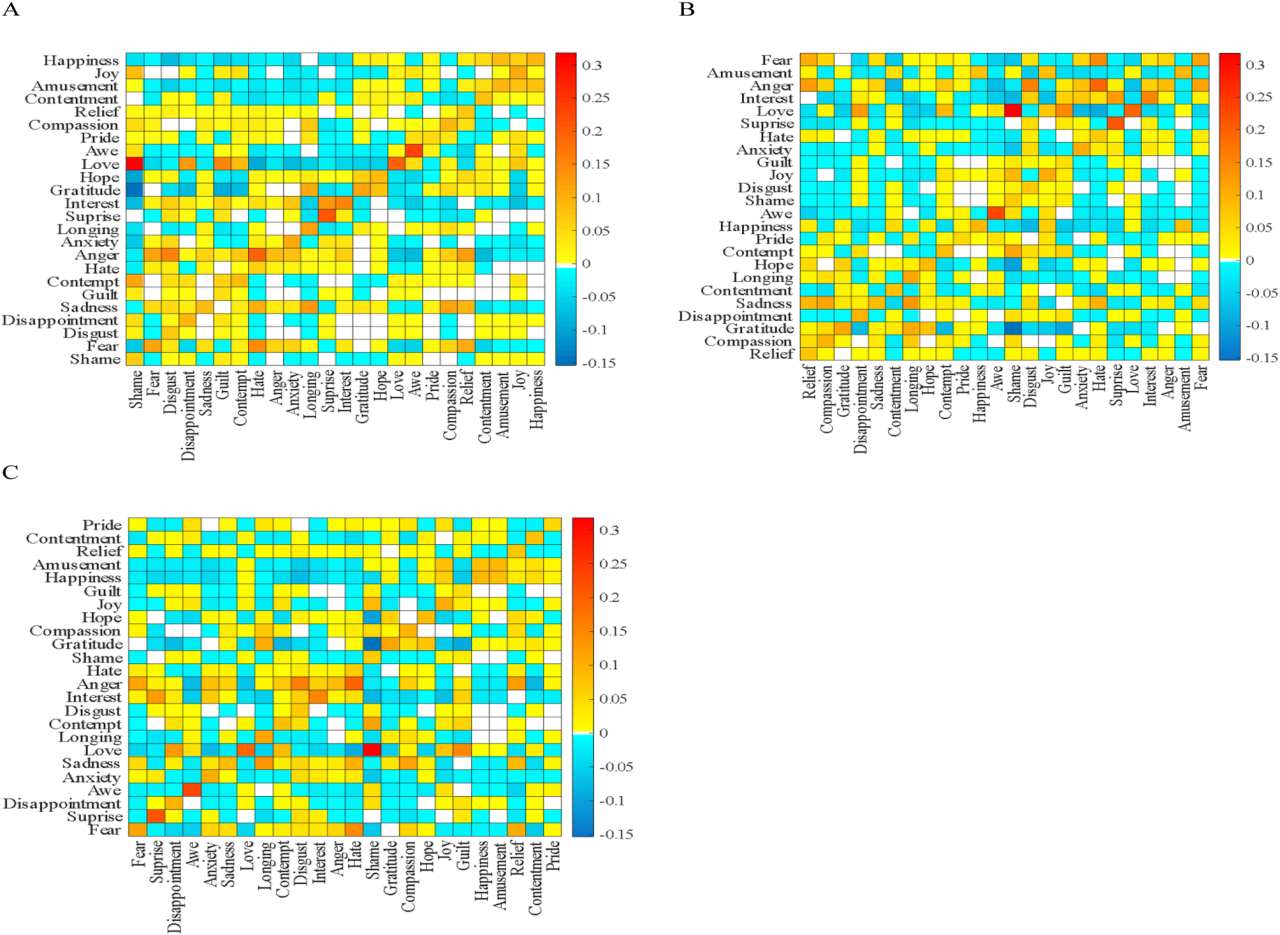
CIRFs capturing long-term transitions. Emotions are positioned in ascending order from bottom-left corner for each dimension: valence (**A**), arousal (**B**), and dominance (**C**). Each cell in the matrix represents how much more (or less) an emotion on the corresponding row will occupy the OSN conversations when the emotion in the corresponding column experiences an exogenous shock. Warm (cold) colors indicate that the emotion on the corresponding row will occupy the conversations more (less). The heatmap was created using heatmap package in MATLAB R2020a version (https://www.mathworks.com/help/matlab/ref/heatmap.html).

**Figure 3:**
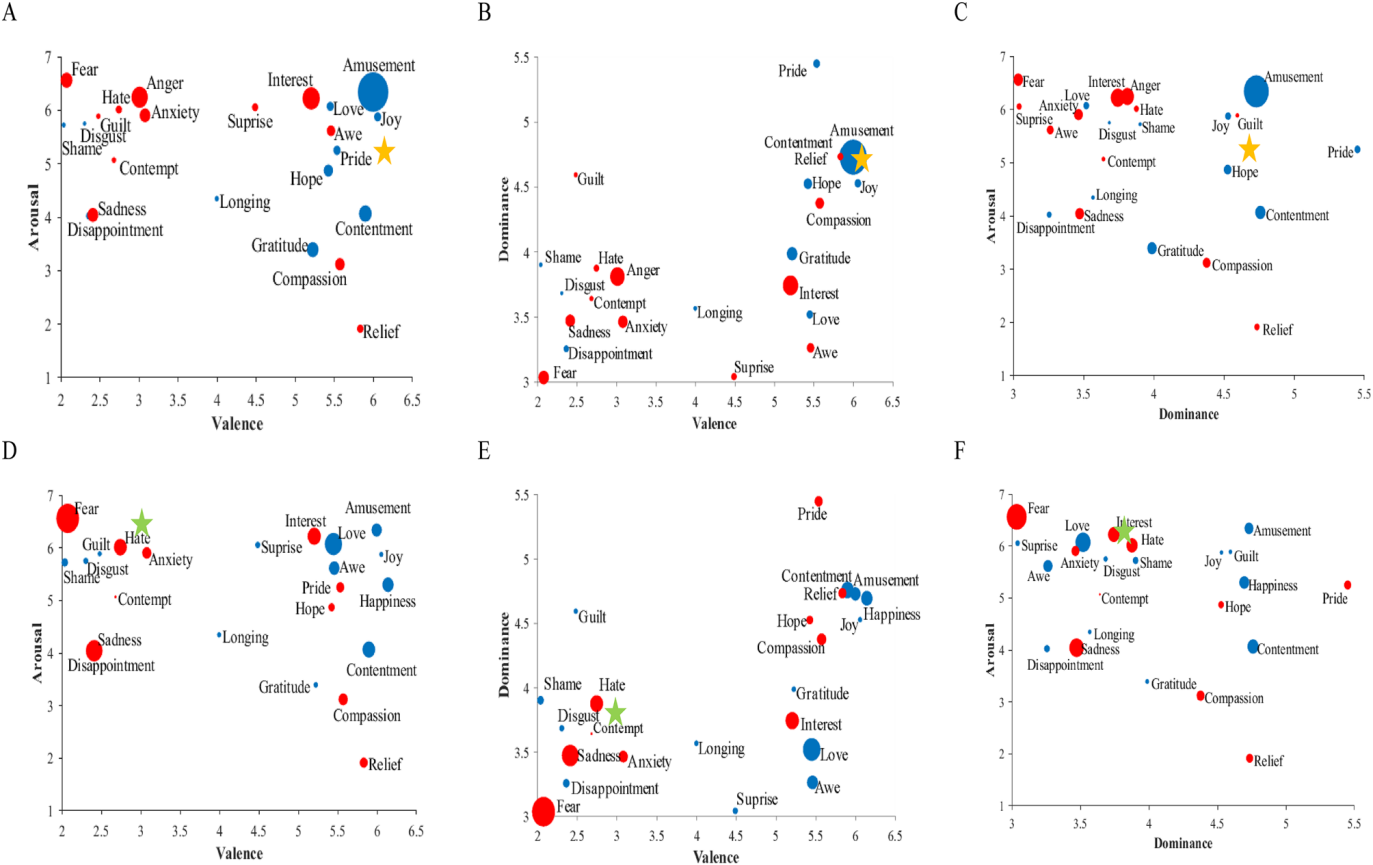
Long-term transitions to all destination emotions in response to a 10% exogenous shock to happiness (top row) and anger (bottom row). Emotions are positioned in the two-dimensional spaces by pairing valence, arousal, and dominance. The bubbles are proportional to the magnitude of the effect, with blue (red) colors indicating an increase (a decrease) in OSN conversations. The yellow (green) star marks the location of the source emotion happiness (anger).

First, we show how an unexpected increase in each emotion affects how much other emotions are expressed in the long run in two ways. Using heatmaps, Fig. 2 depicts the transition likelihood of emotions across three dimensions. According to the observed pattern, emotions that are closer in valence have a higher likelihood of long-term transition, as indicated by a higher number of warmer colors that are closer to the diagonal. In Fig. 3, we show the incremental changes in all destination emotions after an exogenous shock to two exemplary source emotions, happiness, and anger, across the VAD space. The figure, for example, suggests that amusement, which is close to happiness in terms of each dimension, increases the most.

Second, we regressed the CIRFs on the asymmetric distance between emotion pairs to investigate the relationship between emotion dimensions and long-term transitions (Supplementary Table S8, panel labeled CIRF Regression). We find that (1) long-term transitions to destination emotions close to the source emotion on valence dimension are greater than those to distant destination emotions (${\mathrm{CIRF}}_{\mathrm{C}}$ = 0.012999, ${\mathrm{SE}}_{{\mathrm{CIRF}}_{\mathrm{C}}}$ = 0.003799, ${\mathrm{P}}_{{\mathrm{CIRF}}_{\mathrm{C}}}$ < 0.001; ${\mathrm{CIRF}}_{\mathrm{V}}^{+}$ = − 0.002960, ${\mathrm{SE}}_{{\mathrm{CIRF}}_{\mathrm{V}}^{+}}$ = 0.001533, ${\mathrm{P}}_{{\mathrm{CIRF}}_{\mathrm{V}}^{+}}$ = 0.054; ${\mathrm{CIRF}}_{\mathrm{V}}^{-}$ = − 0.002558, ${\mathrm{SE}}_{{\mathrm{CIRF}}_{\mathrm{V}}^{-}}$ = 0.001533, ${\mathrm{P}}_{{\mathrm{CIRF}}_{\mathrm{V}}^{-}}$ = 0.096) and (2) the long-term transitions are independent of the arousal distance (${\mathrm{CIRF}}_{\mathrm{A}}^{+}$ = − 0.002504, ${\mathrm{SE}}_{{\mathrm{CIRF}}_{\mathrm{A}}^{+}}$ = 0.001792, ${\mathrm{P}}_{{\mathrm{CIRF}}_{\mathrm{A}}^{+}}$ > 0.100 and ${\mathrm{CIRF}}_{\mathrm{A}}^{-}$ = − 0.001070, ${\mathrm{SE}}_{{\mathrm{CIRF}}_{\mathrm{A}}^{-}}$ = 0.001792, ${\mathrm{P}}_{{\mathrm{CIRF}}_{\mathrm{A}}^{-}}$ > 0.100) and the dominance distance (${\mathrm{CIRF}}_{\mathrm{D}}^{+}$ = − 0.001168, ${\mathrm{SE}}_{{\mathrm{CIRF}}_{\mathrm{D}}^{+}}$ = 0.003762, ${\mathrm{P}}_{{\mathrm{CIRF}}_{\mathrm{D}}^{+}}$ > 0.100 and ${\mathrm{CIRF}}_{\mathrm{D}}^{-}$ = − 0.005860, ${\mathrm{SE}}_{{\mathrm{CIRF}}_{\mathrm{D}}^{-}}$ = 0.003762, ${\mathrm{P}}_{{\mathrm{CIRF}}_{\mathrm{D}}^{-}}$ > 0.100) between emotion pairs. These findings imply that the carried-over contemporaneous effect (i.e., co-occurrence) overwhelms short-term suppression and that duration differences are not substantial enough to tip the balance, resulting in long-term transition patterns that resemble those observed in co-occurrence.

## Discussion

### Summary

Analyzing millions of social media posts retrieved from Twitter and processed with NLP techniques, this study investigated (a) how much specific emotions occupy OSN conversations, (b) how long they last, and (c) how they follow each other in short- and long-term timeframes and expressed these quantities of interest as a function of three higher-order appraisal dimensions (i.e., valence, arousal, and dominance). We discovered that emotions with lower valence but higher dominance and/or arousal are more likely to occupy OSN conversations; and that the duration of an emotion’s occupation of OSN conversations increases with valence and arousal but decreases with dominance. When an emotion takes over OSN conversations, it suppresses others with similar valence and dominance and higher arousal notably in the short term. In the long run, emotions with similar valences are more likely to follow-up and occupy OSN conversations.

### Contributions

To the best of our knowledge, this study is the first to explore the dynamics of a very large and fine-grained set of emotions and describe these dynamics over an exhaustive set of underlying dimensions. Uncovering the dynamic flow of emotions in OSNs may have important implications for political, economic, and societal outcomes. The role of emotions has been explored in various domains, such as news consumption^55^, false stories^56^, and political content^11^, demonstrating which emotional content spreads more or quickly. Our findings on the evolution of emotions over time can be leveraged to create communication interventions for responding to rising emotional trends effectively. For instance, in times of public health emergencies^57^ or cascading collective traumas^58^, people may experience and express heightened anxiety along with other emotions on social media. Policymakers can benefit from an understanding of the duration of these emotional responses, as well as the potential for subsequent emotions, and finetune their communication strategies. Such informed interventions can help mitigate the negative impacts that exposure to specific emotions through social media can have on public welfare.

Our findings also contribute to the literature by highlighting the role of an overlooked dimension that distinguishes between emotions: dominance. Composed of certainty, agency, and dominance, this higher-order dimension appears as a key descriptor of the dynamic flow of emotions. As evidenced by the differences between low- vs. high-scoring emotions’ 90%-duration intervals, its greatest impact is felt on how long emotions linger in OSN conversations. Dominance is also associated with which other emotions arise as the dynamic flow unfolds. Consider pride, hope, and awe, three emotions that score similarly on valence and arousal but quite different from one another on dominance. Our findings suggest that OSN conversations featuring pride, the emotion with the highest dominance score among the twenty-discrete emotions in this study, will die out much faster than the other two. Assuming away the minor differences in valence and arousal scores of these three emotions, our findings also indicate that awe will be more prevalent in OSN conversations than hope as pride’s grip starts to loosen. Whereas one may postulate why and how the underlying dimensions of valence and arousal are associated with the components of the dynamic flow of emotions in the way they are, our current understanding of dominance prevents us from developing that logic. We believe this represents a fruitful avenue for future research.

## Limitations and avenues for future research

Although the broad range of cognitive appraisal dimensions considered in this study allowed us to identify the novel association between dominance and the dynamic flow of emotions, the list is by no means complete. Consider individuals regulating self-focused emotions (e.g., sadness) or self-conscious emotions (e.g., shame). The potential differences in their use of OSNs—narrowcasting (i.e., communicating with only one person) rather than broadcasting (i.e., communicating with many people) their emotional experiences^59^—implies that the frequency with which certain emotions are expressed in OSN conversations, how long they appear to last, and where they may transition may vary along dimensions other than the three dimensions we investigated. Therefore, future research could explore how other emotion categorizations describe their dynamic flow in OSN conversations.

While this study advances our knowledge of the evolution of emotions in social networks, it is important to note that it does not explore the role of social network structures and user characteristics on the results or distinguish the individual vs. collective nature of emotions and disentangle the mechanisms that give rise to them. The structure of networks can influence the diffusion of behaviors and mental states, such as depression^60^, obesity^61^, exercising^62^, and high-risk movements^63^. Future research could examine how social network structures impact the persistence and transformation of emotions such as pride, gratitude, and contentment, which are known to benefit from social support^64^. In addition to network structures, social network members play a critical role in the spread of behaviors or products. Gender and age have been found to be significant factors in susceptibility and influence within social networks^65^. Demographics may also impact the expression and evolution of emotions online, with men tending to display more assertiveness or dominance-related emotions and women showing more nurturing or empathy-related emotions^66^. Additionally, the presence of strong ties and intergroup conflicts in social networks may serve as predictors of emotional evolution. Research has shown that strong ties are instrumental in spreading both online and real-world behavior in human social networks^67^, while the outgroup effect has been found to be a stronger predictor of social media sharing than emotional language^68^. Taken together, these findings suggest that emotions related to outgroups may lead to greater conflict and prolonged durations, whereas emotions related to ingroup identity may promote emotional convergence and shorter durations within the group.

Moreover, this study describes the dynamics of an aggregation of individually expressed emotions. These individuals are members of a society with a history of common experiences and collective memories, socially shared cognitive appraisal structures, and similar evaluative attitudes, appraising the emotion eliciting events of their everyday lives in relation to their overlapping private concerns or socially grounded shared concerns^69,70^. As such, this study depicts the dynamics of moderately collective (i.e., weak we-mode) emotions^69^ at best. However, there are situations where collective emotions may be more prevalent, such as appraising a singular emotion eliciting event relevant to a preexisting group of individuals with sense of collective commitment^71–73^. Future research should investigate whether and how the dynamics of strongly collective emotions in social networks (i.e., strong we-mode emotions) differ from those identified in this study.

In this study, we investigate the dynamic flow of emotions using daily time-series data obtained by aggregating over emotions extracted using dictionary-based methods from posts on the microblogging site Twitter originated from Turkey-based IP addresses. Future research should pay attention to the various challenges posed by these choices. First, there are alternative approaches to extracting emotions from online text: bottom-up (i.e., machine learning methods) and top-down approaches (i.e., dictionary-based methods). Although there is no single best method that predicts sentiments well, as demonstrated in the context of consumer mindset metrics^74^, bottom-up methods require large training data that is already classified^75^ and perform better in domains in which they are developed^76^. Dictionaries, on the other hand, are transparent and easier to apply. As the constructs (i.e., discrete emotions) were clearly defined and their operationalizations were known, we opted for a top-down approach in this study following^77^. The lack of off-the-self dictionaries for the twenty-four emotions prompted us to develop them from scratch. Creating non-English dictionaries contributes to the establishment of global scientific work in emotion extraction directly and indirectly via the possibility of using their output as features in ensemble machine learning applications to improve predictions^78–81^. Yet, future research might investigate various analysis techniques and compare which ones are more effective and efficient in the extraction of a fine-grained set of emotions. Moreover, we investigate the durations of and transitions among emotions using daily time-series. Performing similar analysis on more finely sliced time-series data (e.g., hour or minute scale) may uncover a richer set of insights.

Second, this study exemplifies the power of emotion-tracking on platforms like Twitter that offers immense potential for understanding large-scale societal trends. Yet, building upon this approach, one must be conscious of the ethical implications and concerns associated with monitoring emotions at a large-scale on a platform such as Twitter. This study presents the emotional dynamics within a society at an aggregate level and does not intend to identify or target specific individuals' or groups of individuals’ emotional changes over time. Thus, findings drawn from Twitter analysis should not be generalized across all segments of society, as this may lead to misrepresentation and further marginalize underrepresented groups.

Third, some emotions might not be freely expressed within social network conversations or not be true reflections of one’s emotions due to various reasons such as social desirability bias and limited cognitive capacity of social media users. Such a challenge holds true for the most observational data retrieved from social networks (e.g., Ref.^8^), and further investigations should explore the aggregate-level dynamics of such emotions using different emotion extraction techniques or data from other sources.

Finally, different social media platforms may have their unique emotional cultures (e.g., a culture of hostility or solidarity^82^) and this may alter the patterns uncovered in this study. We believe this is not a serious concern, as aggregate level public mood time series inferred from Twitter data has been shown to track mood measured by traditional survey methods^83,84^. Yet, it may be a worthwhile endeavor to replicate our analysis using data obtained from different social media platforms. Related, different societies may be operating under different emotional climates (e.g., a climate of fear or hope) influenced by their political and democratic environments^82^. Future research should explore whether and how this characterization of the dynamic flow of emotions varies across different societies with different emotional climates.

### Supplementary Information

Supplementary Information.

## Data Availability

The project has received IRB ethical approval (SBS-2023-39). The Twitter content data that support the findings of this study are publicly available from Twitter but cannot be distributed by the authors due to the risk of inadvertent disclosure of identifiers and ethical issues arising from associating research findings to specific individuals or entities. Daily aggregated data is available from the corresponding author on reasonable request. Survey results and emotion dictionaries are available in the OSF repository (https://osf.io/mt76f/?view_only=4fdcc50f5969460dbae0ef7ca0002779).
