# Supplementary material for: Unveiling the dynamics of emotions in society through an analysis of online social network conversations
Source: Sci Rep. 2023 Sep 11;13:14997. doi: 10.1038/s41598-023-41573-9 (PMC10495421; doi:10.1038/s41598-023-41573-9)
Supplement: Supplementary file 1 — Supplementary Information. [file 41598_2023_41573_MOESM1_ESM.pdf]

UNVEILING THE DYNAMICS OF EMOTIONS IN SOCIETY THROUGH AN ANALYSIS OF ONLINE SOCIAL NETWORK CONVERSATIONS

Begum Sener*
McGill University

Ezgi Akpınar
Sabancı University

M. Berk Ataman
Özyeğin University

Supplementary Information (SI)

Methods.

Dictionary Construction. We adopted a lexicon-based approach for labeling the tweets and created custom-built emotion dictionaries, as lexica for the emotions used in this study did not exist. The dictionary formation process includes the following steps: (i) creating seed dictionaries, (ii) expanding the seed dictionaries using various other validated lexica, (iii) further extending the dictionaries based on human-coded sub-sample of tweets, and (iv) populating dictionaries by adding word derivations.

First, we formed the seed dictionaries using the words in the definitions of each emotion and their synonyms (Table S1). Second, we extended the seed dictionaries by adding related words from wordtracker.com, wordnet.princeton.edu, reversedictionary.org, and relatedwords.org as well as four externally validated lexica: ANEW, GALC, POMS and LIWC. ANEW (Affective Norms for English Words), is a dictionary in which each word has ratings on five specific emotions, namely happiness, anger, fear, disgust, sadness (Stevenson et al., 2007). We used the Turkish version consisting of 2031 words (Kapucu et al., 2021). GALC (Geneva Affective Label Coder) consists of 267 stem words that are categorized into 36 affective states (e.g., amusement, pride, happiness, love, awe, relief, surprise, sadness, shame, disgust, contempt, anger, interest, pride, longing, anxiety, disappointment) (Scherer, 2005). The entries in this dictionary were translated to Turkish and placed into our emotion dictionaries after reaching a consensus among three researchers. POMS (Profile of Mood States) consists of 65 adjectives belonging to six subscales: depression, tension-anxiety, anger, confusion, bewilderment, fatigue, vigor-activity (McNair et al., 1992). We used the Turkish version of POMS (Selvi et al., 1970) and matched the words to the corresponding emotions. Once again, consensus among three researchers was used. Finally, LIWC (Linguistic Inquiry and Word Count), which includes 8202 words and their associations with 64 dimensions was used (Pennebaker et al., 2015). From LIWC, we focused on the words labeled as capturing anxiety, anger, sadness, affect, positive affect, and negative affect. Three researchers reached a consensus about which words to include and where in the twenty-four emotion dictionaries. In all these discussions, the researchers relied on the definitions of the emotions defined in the literature, as well as the context in which the words were used in tweets.

Third, we added unigrams and bigrams related to each emotion extracted from human-coded tweets. We took a random sample of 17,700 tweets from the entire dataset and recruited 100 independent coders to tag the tweets. They were asked to indicate whether the tweet contained any of the twenty-four emotions. As one tweet may contain multiple emotions at the same time or none, choosing more than one emotion or no emotion was allowed. On average, each tweet was tagged by 2.2 coders. 4,796 tweets were tagged by only one coder and one tweet was tagged by seven coders. We used the majority rule (i.e., more than 50% of coders had to agree) to determine which emotion was present in the tweet. Consequently, the tweets that were tagged only once had to be excluded from the sample. For the tweets that were tagged twice (n = 5,434), we retained the emotion tag(s) if both coders agreed on the emotion(s). Of the 8,566 tweets on which the coders agreed, 3,448 contained no emotion and were excluded from further analysis. Each emotion had, on average, 265 tagged tweets and a tweet contained 1.24 emotions on average. With these tagged tweets, we enriched the lexicon via the following steps. Each tweet was first normalized and then tokenized. Next, special words (i.e., hashtags, mentions, and links) were removed. We then lemmatized each word and removed the stop-words with the help of Zemberek-NLP, which provides Natural Language Processing tools for Turkish (https://github.com/ahmetaa/zemberek-nlp/tree/master/morphology). Using the remaining lemmas, we obtained frequency distributions of unigrams and bigrams. Three researchers then went over the list entry by entry to decide whether to include a unigram or a bigram in the corresponding emotion dictionary. Disagreements were solved through multiple rounds of discussion. The last step in the dictionary construction process was to populate the final version of the lexicon with word derivations. As Turkish is an agglutinative language, derivational suffixes can change the meaning or the polarity of a word quite dramatically, hence its inclusion in a specific emotion dictionary. In this step, we benefited from the rather substantial database of zargan.com.

Tweet Scoring Process via Automated Text Analysis. To determine whether a tweet contains specific emotions and, if so, which emotions, we used automated text analysis. The tweets went through the same preprocessing stages as in the enrichment of lexica with the tagged tweets. Specifically, each tweet was normalized, tokenized, and hashtags, links and mentions were removed. The remaining tokens were then lemmatized at the same level as the entries in the dictionaries and stop-words were removed. The 24 emotion dictionaries are then searched for each pre-processed token (called a "unigram") and each pair of tokens that come right after each other (called a "bigram") in a tweet. If a unigram (or bigram) matched an entry in an emotion dictionary, the counter for that emotion was incremented by one, giving us a count of unigrams and bigrams matching an emotion in the tweet. If a tweet has at least one word from the corresponding dictionary, it is assumed to carry that particular emotion.

Obtaining Daily Emotion Time Series. We aggregated the automatically tagged tweets to obtain the daily time series of the twenty-four emotions. The daily expression level of an emotion was calculated by counting the number of tweets containing emotion i posted on day t and dividing this number by the total number of tweets posted on day t, giving us the relative frequency of emotion i on day t (see Figure S1, for time series of each emotion). We checked the performance of the custom-built dictionaries qualitatively by studying the behavior of the time series on important days. Important events affecting the society as a whole can be seen as spikes in the graphs. As expected, January 1 of 2017 and 2018 correspond with peaks in *hope*. Events such as the Atatürk Airport Attack on June 29, 2016, or the Istanbul Beşiktaş Vodafone Stadium Bombing on December 10, 2016, are reflected as spikes in *fear*. *Love* peaks on Valentine's Day; *longing* on Memorial Day (November 11, 2016, and November 11, 2017); *surprise* on April Fools' Day, *pride* on the day after the coup d'état attempt (July 15, 2016), on Victory Day (August 30) and Republic Day (October 29). *Shame* exhibits jumps on dates when news about child sexual abuse hits the nation and discussions around the topic are held in parliament (April 5, 2016, and November 18, 2016).

Dictionary Validation. For validation purposes, we used the NRC (National Research Council Canada) Dictionary (Mohammad & Turney, 2013). The NRC dictionary contains 8,202 words mapped to eight basic emotions (anger, fear, anticipation, trust, surprise, sadness, joy, and disgust) and two sentiments (negative and positive). We used the six emotions in the NRC dictionary that overlap with the emotions in the current study for dictionary validation: namely anger, disgust, fear, joy, sadness, and surprise. We processed the Twitter dataset for a second time using the NRC dictionary and calculated daily relative emotion frequencies (Figure S2). Several differences and commonalities are noteworthy. The first difference is the emotions' base levels of expression across the two dictionaries. Time series obtained using the NRC dictionary have higher base expression levels, possibly because NRC includes generic words such as peace, mother, and love for *joy*. Such words and/or their derivations are, in fact, used to express other emotions in the Turkish language. For example, the word "love" is included in our love dictionary and the word "lovely" is included in our contentment dictionary. Yet, "peace" and "peaceful" are both included in our relief dictionary. Having generic words, which may be used to express emotions not considered in NRC, boosts the daily expression levels of emotions. Another caveat of this is that the time series obtained using the NRC dictionary may have false positives. As examples, *anger* peaks on International Women's Day (March 8, 2017, and March 8, 2018) or *fear* peaks on Mother's Day (May 17, 2017). However, the correlations between the time series obtained by our dictionaries and the NRC dictionaries are positive and reasonably strong: 0.79, 0.53, 0.78, 0.36, 0.80, and 0.45 for anger, disgust, fear, joy, sadness, and surprise, respectively. Collectively, these observations suggest that the custom-built dictionaries do a reasonable job, if not better, in capturing the emotions expressed in the OSN.

Survey.

Participants. 233 college students from a Turkish university participated in the survey for partial course credits. One participant was removed because they took more than one hour to complete the survey. A further 13 participants were removed, leaving us with 219 participants for analysis, because they did not give consent or failed the attention check question.

Procedure. The participants were asked to complete an online survey (in Turkish) about specific emotions and their underlying appraisal dimensions. The surveys were administered to the participants in a computerized lab, under the guidance of the lab administrators using a standardized set of instructions. Each participant was asked to rate seven emotions in total: one trial emotion – selected randomly from a set of six additional emotions – to familiarize the participants with the items in the survey and six emotions randomly drawn from the 24 emotions included in this study. The participants rated each emotion on nine dimensions. The dimensions were presented to the participants in a random order. In total, the participants rated 24 items, one of which was an attention check question, using a 7-point Likert scale (see Table S4, for the items included in the survey). Items used in the survey are adapted from (Mehrabian & Russell, 1974), (Cowen & Keltner, 2017), (Frijda et al., 1989), and (Smith & Ellsworth, 1985). After removing the data that belongs to the trial emotion and the attention check question, we have 1314 emotion-participant ratings.

Analysis*.* To determine the number of underlying dimensions empirically, we resort to exploratory factor analysis. Conventional exploratory factor analysis treats all data points as if they come from different individuals. However, because our data has a nested structure, as multiple participants rate each emotion, multilevel factor analysis is needed. When the nesting structure in the data is not considered, inter-item correlations are over-estimated and standard errors become misleading (Muthén, 1994). Reise et al. (2005) gives an example of data structure which results in different correlation matrices depending on whether within or between group variances are analyzed. Let's assume that a researcher collects dimension ratings from individuals for five emotions. There are ten dimensions to be measured and 24 items in total. Overall, this means that each participant will provide 120 data points: 24 data points for each of the 5 emotions. The researcher wants to investigate the underlying factor structure of the emotions. Consider two dimensions: valence and dominance. On a scale ranging from 1 to 7, where 1 (7) indicates negative (positive) valence and submissiveness (dominance). As it is illustrated, the data structure may be such that (i) for each individual, valence score decreases as dominance score increases, and (ii) between individuals, relationship between valence and dominance is positive. Since in our study we are interested in emotional differences rather than variation within individuals, we looked at between correlation. Therefore, the total correlation matrix needs to be deconstructed into within and between variance matrices. Whereas between variance shows the relationships among the means of measured groups, within variance shows the relationships among the variables within individuals. Since we are interested in differences in emotions, using between group variance is appropriate.

Accordingly, we calculated the between-emotions covariance matrix (S_B) using

(1) $$S_B = \frac{\sum_{i=1}^{I} J_i(\overline{x_i} - \overline{x})(\overline{x_i} - \overline{x})'}{(I-1)}$$

where I is the number of individuals, J_i is the total number of observations within an individual, and $\overline{x}$ is a vector of grand mean. $\overline{x_i}$ indicates the vector of means for individual i. We obtained the between-emotions correlation matrix, R_B (Table S5), by dividing the terms with the appropriate standard deviations and conducted exploratory factor analysis in SPSS. Conventional exploratory factor analysis uses Kaiser's "eigenvalues greater than 1" rule to determine the number of factors. Instead, we opted for Horn's Parallel Analysis (Horn, 1965; Keeling, 2000). Horn's Parallel Analysis procedure compares eigenvalues obtained from the sample to the mean eigenvalues from a sample that is randomly generated via Monte Carlo simulations. Horn's procedure revealed that a three-factor solution is appropriate. The three factors are combinations of (i) valence, time, and the inverse of motivation, inverse of attention, and inverse of effort, (ii) dominance, certainty, and the inverse of agency, and (iii) arousal. See Table S2 for emotions' valence, arousal, and dominance scores.

To validate our findings in relation to the dimensions, we examined the existing literature. The dimension scores we found parallel those in the literature. For example, similar to our findings, contentment and happiness are emotions that score high on valence, whereas sadness and shame score low (Scherer, 2005). Interest is an example of an emotion that deserves attention but is not demanding, appraisal dimensions that make up our valence dimension, whereas shame is an example of an emotion that scores low on those dimensions (Smith & Ellsworth, 1985). Consistent with the literature, fear and anger are high arousal emotions, and sadness and contentment are low arousal emotions (Russell, 1980). Finally, fear and shame are emotions that score low on dominance, whereas contentment and pride score high on dominance (Fontaine et al., 2007). Pride and guilt are examples of emotions that score high on feeling certain about what happened, a part of our dominance dimension, whereas anger and surprise score low on this dimension (Smith & Ellsworth, 1985).

Granger Causality Test.

Before estimating the VAR model, we conducted Granger Causality tests (Granger, 1969) to assess whether a VAR is appropriate to model the dynamics of all twenty-four emotion time series. The Granger Causality test relies on a time series' ability to predict the future behavior of another. Accordingly, causality is based on a notion of incremental predictability and is not concerned with the direction of the relationship (i.e., whether Emotion A increases or decreases Emotion B). Emotion A is said to Granger-cause Emotion B if future observations of Emotion B can be better predicted by a model that uses the past values of Emotion A and Emotion B together, rather than the past values of Emotion B alone. As such, it allows us to see which emotions' past values significantly predict the future values of which other emotions. We performed the test based on a VARX(1) model including all twenty-four emotions in MATLAB.

Based on the results of the Granger Causality tests, we constructed an adjacency matrix. If an emotion is found to Granger-cause another emotion, we set the corresponding element in the adjacency matrix to 1. If not, the corresponding element in the adjacency matrix is set to 0. Using this adjacency matrix, we drew a directed network graph (Figure S3). A review of the directed network graph and the underlying adjacency matrix leads to several observations. First, we observe that causality is predominantly unidirectional. Second, we find that an emotion Granger-causes 2.5 emotions on average, with a minimum of 0 and a maximum of 8 emotions. Third, all twenty-four emotions Granger-cause or are Granger-caused by other emotions, justifying the use of a VAR model.

Model.

The final VAR model, assuming a three-dimensional valence, arousal, and dominance space, is as follows:

$$
\begin{aligned}
\begin{bmatrix} E_t^1 \\ E_t^2 \\ \vdots \\ E_t^{23} \\ E_t^{24} \end{bmatrix} = {} & \mu_0 + \mu_V \begin{bmatrix} V_1 \\ V_2 \\ \vdots \\ V_{23} \\ V_{24} \end{bmatrix} + \mu_A \begin{bmatrix} A_1 \\ A_2 \\ \vdots \\ A_{23} \\ A_{24} \end{bmatrix} + \mu_D \begin{bmatrix} D_1 \\ D_2 \\ \vdots \\ D_{23} \\ D_{24} \end{bmatrix} + \mu_S \begin{bmatrix} S_1 \\ S_2 \\ \vdots \\ S_{23} \\ S_{24} \end{bmatrix} + \lambda_0 \begin{bmatrix} E_{t-1}^1 \\ E_{t-1}^2 \\ \vdots \\ E_{t-1}^{23} \\ E_{t-1}^{24} \end{bmatrix} + \lambda_V \begin{bmatrix} V_1 E_{t-1}^1 \\ V_2 E_{t-1}^2 \\ \vdots \\ V_{23} E_{t-1}^{23} \\ V_{24} E_{t-1}^{24} \end{bmatrix} \\
& + \lambda_A \begin{bmatrix} A_1 E_{t-1}^1 \\ A_2 E_{t-1}^2 \\ \vdots \\ A_{23} E_{t-1}^{23} \\ A_{24} E_{t-1}^{24} \end{bmatrix} + \lambda_D \begin{bmatrix} D_1 E_{t-1}^1 \\ D_2 E_{t-1}^2 \\ \vdots \\ D_{23} E_{t-1}^{23} \\ D_{24} E_{t-1}^{24} \end{bmatrix} + \lambda_S \begin{bmatrix} S_1 E_{t-1}^1 \\ S_2 E_{t-1}^2 \\ \vdots \\ S_{23} E_{t-1}^{23} \\ S_{24} E_{t-1}^{24} \end{bmatrix} \\
& + \tau_0 \begin{bmatrix} 0 + & E_{t-1}^2 + & \cdots & + E_{t-1}^{23} + & E_{t-1}^{24} \\ E_{t-1}^1 + & 0 + & \cdots & + E_{t-1}^{23} + & E_{t-1}^{24} \\ \vdots & \vdots & \ddots & \vdots & \vdots \\ E_{t-1}^1 + & E_{t-1}^2 + & \cdots & + 0 + & E_{t-1}^{24} \\ E_{t-1}^1 + & E_{t-1}^2 + & \cdots & + E_{t-1}^{23} + & 0 \end{bmatrix} \\
& + \tau_V \begin{bmatrix} 0 + & DIST_{1,2}^1 E_{t-1}^2 + & \cdots & + DIST_{1,23}^1 E_{t-1}^{23} + & DIST_{1,24}^1 E_{t-1}^{24} \\ DIST_{2,1}^1 E_{t-1}^1 + & 0 + & \cdots & + DIST_{2,23}^1 E_{t-1}^{23} + & DIST_{2,24}^1 E_{t-1}^{24} \\ \vdots & \vdots & \ddots & \vdots & \vdots \\ DIST_{23,1}^1 E_{t-1}^1 + & DIST_{23,2}^1 E_{t-1}^2 + & \cdots & + 0 + & DIST_{23,24}^1 E_{t-1}^{24} \\ DIST_{24,1}^1 E_{t-1}^1 + & DIST_{24,2}^1 E_{t-1}^2 + & \cdots & + DIST_{24,23}^1 E_{t-1}^{23} + & 0 \end{bmatrix} \\
& + \tau_A \begin{bmatrix} 0 + & DIST_{1,2}^2 E_{t-1}^2 + & \cdots & + DIST_{1,23}^2 E_{t-1}^{23} + & DIST_{1,24}^2 E_{t-1}^{24} \\ DIST_{2,1}^2 E_{t-1}^1 + & 0 + & \cdots & + DIST_{2,23}^2 E_{t-1}^{23} + & DIST_{2,24}^2 E_{t-1}^{24} \\ \vdots & \vdots & \ddots & \vdots & \vdots \\ DIST_{23,1}^2 E_{t-1}^1 + & DIST_{23,2}^2 E_{t-1}^2 + & \cdots & + 0 + & DIST_{23,24}^2 E_{t-1}^{24} \\ DIST_{24,1}^2 E_{t-1}^1 + & DIST_{24,2}^2 E_{t-1}^2 + & \cdots & + DIST_{24,23}^2 E_{t-1}^{23} + & 0 \end{bmatrix} \\
& + \tau_D \begin{bmatrix} 0 + & DIST_{1,2}^3 E_{t-1}^2 + & \cdots & + DIST_{1,23}^3 E_{t-1}^{23} + & DIST_{1,24}^3 E_{t-1}^{24} \\ DIST_{2,1}^3 E_{t-1}^1 + & 0 + & \cdots & + DIST_{2,23}^3 E_{t-1}^{23} + & DIST_{2,24}^3 E_{t-1}^{24} \\ \vdots & \vdots & \ddots & \vdots & \vdots \\ DIST_{23,1}^3 E_{t-1}^1 + & DIST_{23,2}^3 E_{t-1}^2 + & \cdots & + 0 + & DIST_{23,24}^3 E_{t-1}^{24} \\ DIST_{24,1}^3 E_{t-1}^1 + & DIST_{24,2}^3 E_{t-1}^2 + & \cdots & + DIST_{24,23}^3 E_{t-1}^{23} + & 0 \end{bmatrix} \\
& + \tau_S \begin{bmatrix} 0 + & S_1 E_{t-1}^2 + & \cdots & + S_1 E_{t-1}^{23} + & S_1 E_{t-1}^{24} \\ S_2 E_{t-1}^1 + & 0 + & \cdots & + S_2 E_{t-1}^{23} + & S_2 E_{t-1}^{24} \\ \vdots & \vdots & \ddots & \vdots & \vdots \\ S_{23} E_{t-1}^1 + & S_{23} E_{t-1}^2 & \cdots & + 0 + & S_{23} E_{t-1}^{24} \\ S_{24} E_{t-1}^1 + & S_{24} E_{t-1}^2 & \cdots & + S_{24} E_{t-1}^{23} + & 0 \end{bmatrix} + \begin{bmatrix} \beta_{1,1} \\ \vdots \\ \beta_{24,1} \end{bmatrix} DUM1 \\
& + \begin{bmatrix} \beta_{1,2} \\ \vdots \\ \beta_{24,2} \end{bmatrix} DUM2
\end{aligned}
\tag{2}
$$

where V_i, A_i, and D_i are mean-centered dimension scores of emotion i, S_i is the dictionary size of emotion i, DUM1 and DUM2 are the step dummies, and $DIST_{ij}^k$ is the distance between source emotion j and destination emotion i on dimension k (k=1 for valence, k=2 for arousal, and k=3 for dominance). To allow the direction of the effect to be different when moving up versus moving down along a dimension (i.e., asymmetric directional), we decompose $DIST_{ij}^k$ into two variables:

(3a) $$\mathrm{DIST}_{ij}^{k+} = \begin{cases} \mathrm{DIM}_{ik} - \mathrm{DIM}_{jk}, & \forall\ \mathrm{DIM}_{ik} > \mathrm{DIM}_{jk} \\ 0, & \text{otherwise} \end{cases}$$

(3b) $$\mathrm{DIST}_{ij}^{k-} = \begin{cases} \mathrm{DIM}_{jk} - \mathrm{DIM}_{ik}, & \forall\ \mathrm{DIM}_{ik} > \mathrm{DIM}_{jk} \\ 0, & \text{otherwise} \end{cases}$$

where i and j index emotions (i = 1,2,3,…, 24 and j = 1,2,3,.., 24) and k indexes dimensions (k=1,2,3). DIM_{ik} (DIM_{jk}) represents the score of emotion i (j) in dimension k. In the model that uses the asymmetric and directional distance operationalization, transition coefficients are labeled as τ_k^+ and τ_k^-.

In addition to the asymmetric directional distance used in the main model, we entertained two alternative ways of calculating the distances between emotion pairs: a symmetric directional distance and an asymmetric non-directional distance.

If the effect of movement direction is disregarded and only the distance between emotion pairs is assumed to have an effect (i.e., non-directional), then

(4) $$\mathrm{DIST}_{ij}^{k} = \left|\mathrm{DIM}_{ik} - \mathrm{DIM}_{jk}\right|$$

In the model with the non-directional distance operationalization, transition coefficients are labeled as $\tau_{|k|}$.

If the effect of movement direction is assumed to be symmetrical when moving up and down along a dimension (i.e., symmetric directional), then

(5) $$\mathrm{DIST}_{ij}^{k} = \mathrm{DIM}_{ik} - \mathrm{DIM}_{jk}$$

In the model that uses the symmetric and directional distance operationalization, transition coefficients are labeled as τ_k.

All models are estimated using STATA`s command *xtgls* which applies Generalized Least Squares Regression. Table S7 presents the results based on these operationalizations.

Table S1. List and definitions of emotions

Emotion	Definition
Contempt	An emotion that contains a sense of superiority. It occurs in situations that are viewed as degrading and includes negative judgements about the other person, object, or situation.
Love	An extremely strong sense of attachment to (in)tangible entities, beyond mere liking or enjoyment. People who feel love may have difficulty controlling their emotions and experience confusion.
Contentment	An emotion that arises when a person, object, or situation is evaluated positively or favorably. It is a state of satisfaction and quiet pleasure.
Amusement	An emotion that occurs in funny, exciting, and joyful situations. It includes experiencing fun while laughing at a funny joke, object, or person, or playing a game.
Anxiety	A state of being occupied by suspicious thoughts, such as thinking something bad will happen or fearing being disgraced or ridiculed. It is accompanied by feelings of terror, distress, and tension, and includes physical symptoms, such as sweating, tremors, palpitations, etc.
Pride	An emotion that is felt because of being recognized and approved by others for achieving a goal, succeeding in something, or reaching a desired end-state. One likes and feels good about oneself.
Disappointment	A state of dissatisfaction or sadness caused by the failure of hopes or expectations.
Awe	A feeling that includes pleasure, curiosity, respect for people, objects, and situations that exceed competence, ability, or skill expectations.
Interest	An emotion felt in situations where one’s interest and attention are directed to a person, object, or situation, with heightened concentration, curiosity, and stimulation.
Hate	A strongly repulsive feeling wherein one evaluates a person, object, or situation intensely negatively and wishes their evil or unhappiness.
Fear	A state of helplessness in the face of danger or feeling anxious due to being unable to maintain control.
Gratitude	Appreciation of the kindness, goods, or earnings unconditionally given by someone else.
Happiness	A state of contentment about the current situation and feeling well because of an enjoyable or desired experience that often makes one smile. It is less enthusiastic than the feeling of joy.
Anger	A violent state that can lead to aggression in situations such as injustice, being hurt, not keeping one’s promises, and telling lies.

Table S1 (cont'd). List and definitions of emotions

Emotion	Definition
Longing	A need or desire to meet a person, see an object, or experience a situation once more.
Relief	A state of feeling assured and relaxed after a distressful, anxiety-evoking situation fails to materialize or disappears.
Joy	An enthusiastic, beautiful, and positive state that is felt momentarily, for a shorter period than happiness.
Guilt	A state of feeling uncomfortable and responsible because of having made a mistake or having failed, one that encourages oneself to compensate for the mistake, the failure, or the wrongdoing.
Surprise	A state of wonder in the face of an unexpected and unaccustomed situation.
Compassion	A state that includes empathy, acceptance, tolerance, and motivation to help ease others' pain or distress.
Disgust	A feeling that emerges when confronted with a person, object, or situation that is deemed revolting or repugnant, one that causes a wrinkled face, nausea, or the desire to avoid the stimulus.
Hope	A state of feeling optimistic about the future, wherein one feels that goals and aspirations are attainable and encouraged to persevere for reaching them.
Shame	A state of disaffection arising from falling into a flawed situation or seeing the person, object, or situation as being essentially flawed, accompanied by feelings of embarrassment and weakness.
Sadness	An annoying, painful, and unpleasant emotion felt in situations where the expectation is not realized, or the result is undesirable. It may be accompanied by physical symptoms, such as a desire to cry.

Emotion definitions are adapted from https://dictionary.apa.org/.

Table S2. Descriptive statistics of emotion time series and emotions' dimension scores

	Descriptive Statistics of Emotion Time Series							*Emotions' Dimension Scores*		
Emotion Name	Mean	Std. Dev.	CV	Kurtosis	Skewness	Minimum	Maximum	Valence	Arousal	Dominance
Contempt	0.046	0.005	0.110	0.632	0.625	0.033	0.067	2.679	5.067	3.642
Love	0.058	0.008	0.141	12.583	1.240	0.018	0.126	5.446	6.071	3.520
Contentment	0.148	0.015	0.104	1.161	-0.045	0.075	0.199	5.896	4.067	4.760
Amusement	0.067	0.018	0.272	40.602	5.049	0.036	0.247	5.995	6.340	4.730
Anxiety	0.101	0.011	0.106	0.777	0.969	0.078	0.145	3.078	5.903	3.465
Pride	0.051	0.017	0.331	16.623	3.226	0.029	0.195	5.532	5.248	5.451
Disappointment	0.028	0.004	0.129	0.600	0.777	0.020	0.042	2.360	4.024	3.257
Awe	0.031	0.005	0.169	0.730	0.659	0.017	0.055	5.456	5.614	3.264
Interest	0.154	0.013	0.085	0.565	0.862	0.121	0.209	5.202	6.220	3.744
Hate	0.033	0.008	0.252	22.191	3.783	0.021	0.104	2.742	6.012	3.877
Fear	0.047	0.015	0.316	29.740	4.406	0.030	0.191	2.074	6.560	3.036
Gratitude	0.041	0.010	0.234	3.228	1.480	0.026	0.101	5.222	3.393	3.988
Happiness	0.069	0.018	0.267	38.304	4.904	0.030	0.245	6.139	5.298	4.696
Anger	0.149	0.027	0.179	18.146	3.492	0.107	0.346	3.008	6.242	3.812
Longing	0.020	0.005	0.260	14.824	2.893	0.008	0.061	3.995	4.346	3.568
Relief	0.028	0.006	0.199	2.128	1.279	0.018	0.054	5.831	1.915	4.735
Joy	0.016	0.006	0.377	4.132	1.665	0.006	0.049	6.054	5.875	4.530
Guilt	0.019	0.003	0.153	1.056	0.716	0.012	0.034	2.480	5.887	4.595
Surprise	0.024	0.004	0.146	4.607	1.253	0.017	0.047	4.486	6.055	3.042
Compassion	0.025	0.007	0.296	8.827	2.303	0.013	0.079	5.570	3.119	4.377
Disgust	0.015	0.002	0.164	1.355	0.775	0.008	0.026	2.304	5.750	3.685
Hope	0.091	0.014	0.151	3.531	1.465	0.066	0.177	5.423	4.870	4.527
Shame	0.006	0.002	0.273	5.397	1.446	0.002	0.016	2.036	5.721	3.903
Sadness	0.182	0.020	0.108	5.878	1.732	0.138	0.300	2.410	4.042	3.473

Reviewing the descriptive statistics of emotion time series, we observe that, out of all the emotions, sadness ($\mu_{Sadness}$ = .182) has the highest average frequency level followed by interest ($\mu_{Interest}$ = .154), while shame has the lowest average frequency (μ_{Shame} = .006) followed by disgust ($\mu_{Disgust}$ = .015) and joy (μ_{Joy} = .016). Joy, pride, and fear exhibit the greatest relative variability over time (CV_{Joy} = .377, CV_{Pride} = .331, and CV_{Fear} = .316), whereas interest, contentment, and anxiety exhibit the lowest ($CV_{Interest}$ = .085, $CV_{Contentment}$ = .104, and $CV_{Anxiety}$ = .106). Valence score for each emotion is calculated as the average of valance, time, and inverse of motivation, inverse of attention, and inverse of effort dimensions. Dominance score of each emotion is calculated as the average of dominance, certainty, and inverse of agency.

Table S3. Overview of major appraisal dimensions and selected empirical work

Dimension	Definition	Relevant Literature
Valence	Being positive or negative (motive-inconsistent, motive-consistent)	(Mehrabian & Russell, 1974)
Arousal	Level of activation (sleep-tension)	(Mehrabian & Russell, 1974)
Dominance	Being in control or controlled	(Mehrabian & Russell, 1974)
Motivational State	Being in an appetitive or aversive state	(Smith & Ellsworth, 1985)
Time	Whether the event causing the emotion is related to future or past	(Frijda et al., 1989)
Agency	Whether the cause is circumstances, others, or the self	(Smith & Ellsworth, 1985)
Effort	Having to do something or relax	(Smith & Ellsworth, 1985)
Certainty	Being certain or uncertain about what is happening	(Smith & Ellsworth, 1985)
Attention	Motive to keep seeking or to look away	(Smith & Ellsworth, 1985)

Table S4. Items in the survey

Question: If someone from your social environment and from the same cultural background as you describes the emotion they feel as X, …

Dimension	Item	Adapted from
Valence	*…please tell us how this person might be feeling.*	
	Unhappy (1) - Happy (7)	(Mehrabian & Russell, 1974)
	Annoyed (1) - Pleased (7)	(Mehrabian & Russell, 1974)
	Unsatisfied (1) - Satisfied (7)	(Mehrabian & Russell, 1974)
Arousal	*…please tell us how this person might be feeling.*	
	Relaxed (1) - Stimulated (7)	(Mehrabian & Russell, 1974)
	Calm (1) - Excited (7)	(Mehrabian & Russell, 1974)
	Sleepy (1) - Wide Awake (7)	(Mehrabian & Russell, 1974)
Dominance	*…please tell us how this person might be feeling.*	
	Influenced (1) - Influential (7)	(Mehrabian & Russell, 1974)
	Submissive (1) - Dominant (7)	(Mehrabian & Russell, 1974)
	Guided (1) - Autonomous (7)	(Mehrabian & Russell, 1974)
Motivational State	*… please tell us how they might be feeling towards the person, the situation, or the object that caused the emotion.*	
	Desires to approach (1) – Desires to avoid (7) that someone or something.	(Cowen & Keltner, 2017)
	Wants to make contact (1) – Wants to have nothing to do (7) with that someone or something.	(Frijda et al., 1989)
	Wants to be or stay close, to be receptive to (1) – Wants to protect themselves from (7) that someone or something.	(Frijda et al., 1989)
Time	*... they think that this feeling is more related to the ...*	
	Past (1) – Present (4) – Future (7)	(Frijda et al., 1989)
Agency	*… they would think that…* (1: Completely disagree – 7: Completely agree)	
	… they were the one responsible for the event causing them to feel X in this situation.	(Smith & Ellsworth, 1985)
	… someone else was responsible for the event causing them to feel X in this situation.	(Smith & Ellsworth, 1985)
	… it was beyond anyone's control.	(Smith & Ellsworth, 1985)
Effort	*… how much …* (1: Not at all – 7: Extremely)	
	… would they need to exert themselves to deal with this situation?	(Smith & Ellsworth, 1985)
	… physical or psychological effort would this person think they need to exert in this situation?	(Smith & Ellsworth, 1985)
Certainty	*… they probably…*	
	… does not understand (1) – understands clearly (7) what was happening in this situation.	(Smith & Ellsworth, 1985)
	… is not certain (1) – is certain (7) about what was happening in this situation.	(Smith & Ellsworth, 1985)
	… cannot predict (1) – can predict (7) what was going to happen in this situation.	(Smith & Ellsworth, 1985)
Attention	*… they…*	
	… would try to consider this thing further (1) – would try to shut it out (7).	(Smith & Ellsworth, 1985)
	… would try to devote their attention to this thing (1) - would try to divert their attention from it (7).	(Smith & Ellsworth, 1985)

Table S5. Between-correlation matrix

	valence$_1$	*valence$_2$*	*valence$_3$*	*arousal$_1$*	*arousal$_2$*	*arousal$_3$*	*dom$_1$*	*dom$_2$*	*dom$_3$*	*mot1*	*mot2*	*mot3*
valence$_1$	1.000											
valence$_2$	.999	1.000										
valence$_3$	.997	.998	1.000									
arousal$_1$	-.020	-.043	-.072	1.000								
arousal$_2$	-.553	-.571	-.592	.816	1.000							
arousal$_3$	.011	-.017	-.041	.953	.743	1.000						
dom$_1$	.638	.647	.644	.017	-.363	.124	1.000					
dom$_2$	.514	.521	.512	.093	-.212	.203	.908	1.000				
dom$_3$	.632	.641	.634	.021	-.331	.121	.931	.970	1.000			
mot$_1$	-.930	-.931	-.931	.042	.514	.066	-.555	-.435	-.544	1.000		
mot$_2$	-.920	-.921	-.923	.047	.509	.078	-.546	-.416	-.528	.998	1.000	
mot$_3$	-.915	-.917	-.919	.071	.541	.092	-.575	-.441	-.552	.994	.994	1.000
time	.396	.382	.360	.271	-.028	.235	.211	.178	.230	-.352	-.326	-.315
agency$_1$	-.232	-.244	-.246	.016	.172	-.004	-.596	-.393	-.452	.175	.196	.224
agency$_2$	-.214	-.228	-.229	.060	.185	.053	-.531	-.317	-.385	.253	.271	.296
agency$_3$	.183	.190	.175	.173	.062	.009	-.199	-.214	-.106	-.296	-.306	-.295
effort$_1$	-.893	-.898	-.901	.194	.646	.113	-.673	-.620	-.714	.772	.758	.761
effort$_2$	-.851	-.858	-.863	.269	.697	.180	-.673	-.614	-.702	.729	.716	.725
certain$_1$	.463	.474	.489	-.417	-.648	-.289	.606	.499	.525	-.360	-.351	-.404
certain$_2$	.473	.483	.500	-.395	-.630	-.260	.638	.552	.559	-.373	-.360	-.410
certain$_3$	.671	.677	.690	-.333	-.692	-.223	.718	.624	.663	-.588	-.573	-.609
attention$_1$	-.869	-.867	-.867	-.054	.392	-.032	-.609	-.520	-.606	.925	.924	.912
attention$_2$	-.885	-.883	-.882	-.067	.391	-.053	-.618	-.525	-.615	.930	.929	.915

	time	*agency$_1$*	*agency$_2$*	*agency$_3$*	*effort$_1$*	*effort$_2$*	*certain$_1$*	*certain$_2$*	*certain$_3$*	*attention$_1$*	*attention$_2$*
valence$_1$											
valence$_2$											
valence$_3$											
arousal$_1$											
arousal$_2$											
arousal$_3$											
dom$_1$											
dom$_2$											
dom$_3$											
mot$_1$											
mot$_2$											
mot$_3$											
time	1.000										
agency$_1$	.023	1.000									
agency$_2$	-.010	.919	1.000								
agency$_3$	.286	.194	.067	1.000							
effort$_1$	-.261	.151	.095	-.024	1.000						
effort$_2$	-.214	.164	.112	.044	.990	1.000					
certain$_1$	-.141	-.411	-.264	-.315	-.600	-.616	1.000				
certain$_2$	-.159	-.352	-.214	-.340	-.608	-.624	.979	1.000			
certain$_3$	.148	-.288	-.177	-.244	-.786	-.794	.893	.906	1.000		
attention$_1$	-.351	.170	.205	-.161	.721	.683	-.229	-.260	-.490	1.000	
attention$_2$	-.374	.186	.222	-.165	.731	.689	-.233	-.258	-.490	.997	1.000

Table S6. Fit statistics for VAR models with different lag lengths

VAR lag length	Loglikelihood	AIC	BIC
1 lag	**74155.0**	**-147014**	**-143987**
2 lags	74492.9	-146538	-140822
3 lags	74789.1	-145978	-137575

Table S7. Coefficient estimates of the proposed model and its benchmarks

	Proposed Model				Benchmark Models for Sensitivity Checks							
	Directional & Asymmetric				Non-directional & Symmetric				Directional & Symmetric			
	Coef.	SE	P-Value		Coef.	SE	P-Value		Coef.	SE	P-Value	
Frequency												
Intercept (μ_0)	0.017	0.001	0.000	***	0.017	0.001	0.000	***	0.017	0.001	0.000	***
Valence (μ_V)	-0.001	0.000	0.022	**	-0.001	0.000	0.000	***	0.000	0.000	0.392	
Arousal (μ_A)	0.001	0.000	0.065	*	0.000	0.000	0.776		0.002	0.000	0.000	***
Dominance (μ_D)	0.005	0.001	0.000	***	0.005	0.000	0.000	***	0.007	0.001	0.000	***
Dictionary Size (μ_S)	0.000	0.000	0.000	***	0.000	0.000	0.000	***	0.000	0.000	0.000	***
Duration												
Intercept (λ_0)	0.856	0.004	0.000	***	0.855	0.004	0.000	***	0.860	0.004	0.000	***
Valence (λ_V)	0.057	0.002	0.000	***	0.058	0.002	0.000	***	0.043	0.002	0.000	***
Arousal (λ_A)	0.011	0.002	0.000	***	0.010	0.002	0.000	***	0.004	0.002	0.039	**
Dominance (λ_D)	-0.165	0.006	0.000	***	-0.168	0.006	0.000	***	-0.131	0.005	0.000	***
Dictionary Size (λ_S)	0.000	0.000	0.150		0.000	0.000	0.080	*	0.000	0.000	0.000	***
Transition												
Intercept (τ_0)	-0.011	0.001	0.000	***	-0.011	0.001	0.000	***	-0.007	0.001	0.000	***
Directional + Distance on V (τ_V^+)	0.001	0.000	0.000	***								
Directional - Distance on V (τ_V^-)	0.002	0.000	0.000	***								
Distance on V ($\tau_{\lvert V \rvert}$)					0.001	0.000	0.000	***				
Directional Distance on V (τ_V)									0.000	0.000	0.280	
Directional + Distance on A (τ_A^+)	-0.001	0.000	0.000	***								
Directional - Distance on A (τ_A^-)	0.000	0.000	0.915									
Distance on A ($\tau_{\lvert A \rvert}$)					-0.001	0.000	0.000	***				
Directional Distance on A (τ_A)									-0.001	0.000	0.028	**
Directional + Distance on D (τ_D^+)	0.004	0.001	0.000	***								
Directional - Distance on D (τ_D^-)	0.004	0.001	0.000	***								
Distance on D ($\tau_{\lvert D \rvert}$)					0.004	0.000	0.000	***				
Directional Distance on D (τ_D)									-0.001	0.001	0.070	*
Dictionary Size (τ_S)	0.000	0.000	0.000	***	0.000	0.000	0.000	***	0.000	0.000	0.000	***

All regressions are GLS models and are estimated using heteroskedasticity-consistent SEs. Robust p-values and their significance are shown, * p = 10%, ** p = 5%, and *** p = 1%.

Table S8. Contemporaneous Effect (CE) and Cumulative Impulse Response (CIRF) Regressions

CE Regression				
	Coef.	SE	P-value	
Constant ($\mathrm{CE_C}$)	**0.021**	**0.004**	**0.000**	***
Directional + Distance on V ($\mathrm{CE_V^+}$)	**-0.005**	**0.001**	**0.000**	***
Directional - Distance on V ($\mathrm{CE_V^-}$)	**-0.006**	**0.001**	**0.000**	***
Directional + Distance on A ($\mathrm{CE_A^+}$)	-0.002	0.002	0.252	
Directional - Distance on A ($\mathrm{CE_A^-}$)	0.001	0.002	0.434	
Directional + Distance on D ($\mathrm{CE_D^+}$)	-0.004	0.004	0.210	
Directional - Distance on D ($\mathrm{CE_D^-}$)	**-0.008**	**0.004**	**0.023**	**
**CIRF Regression**				
	Coef.	SE	P-value	
Constant ($\mathrm{CIRF_C}$)	**0.013**	**0.004**	**0.001**	***
Directional + Distance on V ($\mathrm{CIRF_V^+}$)	**-0.003**	**0.002**	**0.054**	*
Directional - Distance on V ($\mathrm{CIRF_V^-}$)	**-0.003**	**0.002**	**0.096**	*
Directional + Distance on A ($\mathrm{CIRF_A^+}$)	-0.003	0.002	0.163	
Directional - Distance on A ($\mathrm{CIRF_A^-}$)	-0.001	0.002	0.551	
Directional + Distance on D ($\mathrm{CIRF_D^-}$)	-0.001	0.004	0.756	
Directional - Distance on D ($\mathrm{CIRF_D^-}$)	-0.006	0.004	0.120	

To examine how the contemporaneous effects of 10% exogenous shocks to different source emotions on destination emotions (i.e., co-occurrence effect) vary as a function of asymmetric distances between emotion pairs, we estimate the following regression equation: $\mathrm{CE_{ij}} = \mathrm{CE_0} + \sum_{k=1}^{K} \mathrm{CE_k^+ DIST_{ij}^{k+}} + \sum_{k=1}^{K} \mathrm{CE_k^- DIST_{ij}^{k-}}$. To investigate the relationship between long-term transitions and emotion dimensions, we regress cumulative IRFs on asymmetric distances between emotion pairs using the following equation: $\mathrm{CIRF_{ij}} = \mathrm{CIRF_0} + \sum_{k=1}^{K} \mathrm{CIRF_k^+ DIST_{ij}^{k+}} + \sum_{k=1}^{K} \mathrm{CIRF_k^- DIST_{ij}^{k-}}$. * p = 10%, ** p = 5%, and *** p = 1%.

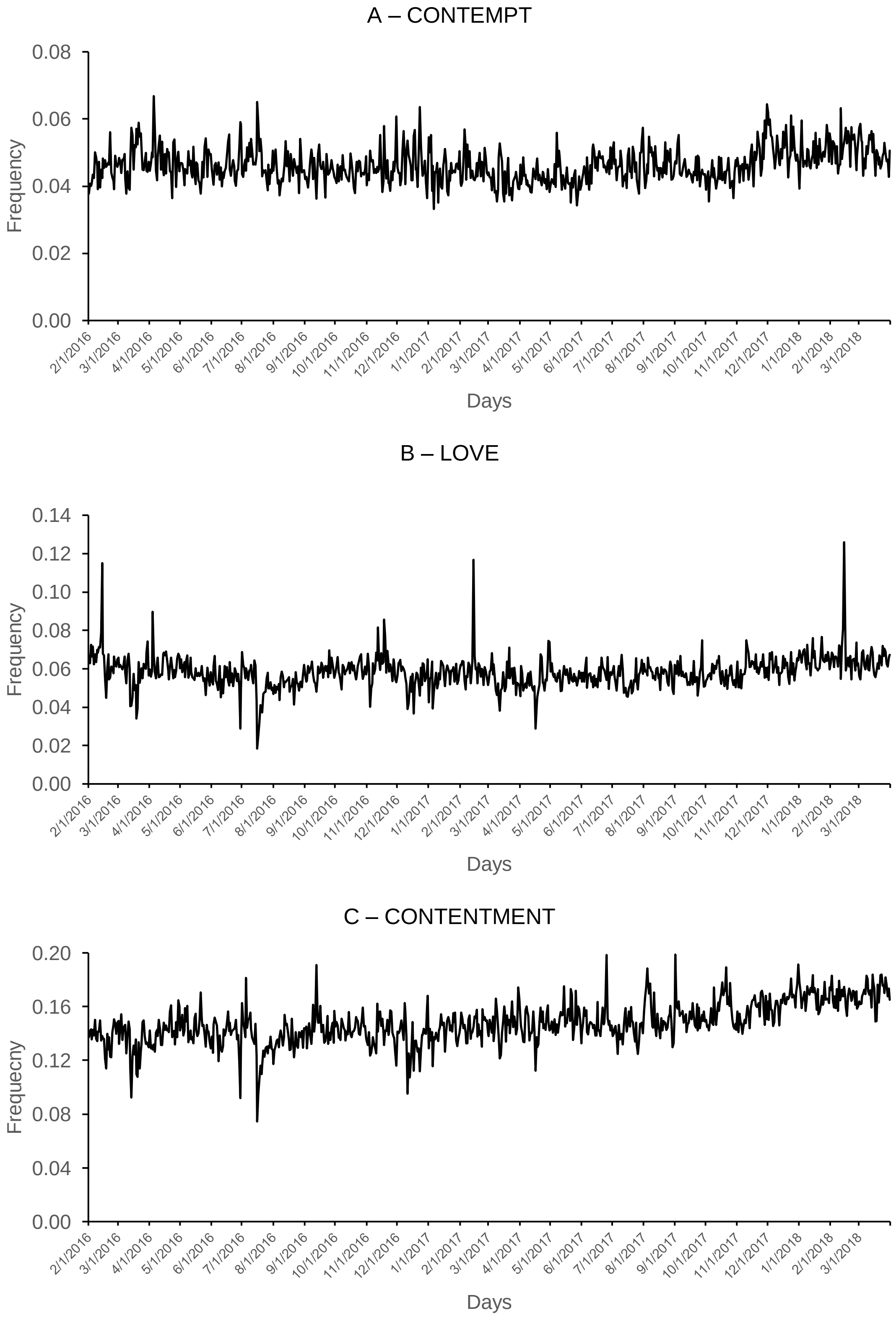

Figure S1. Time series plots of daily frequencies of emotions.

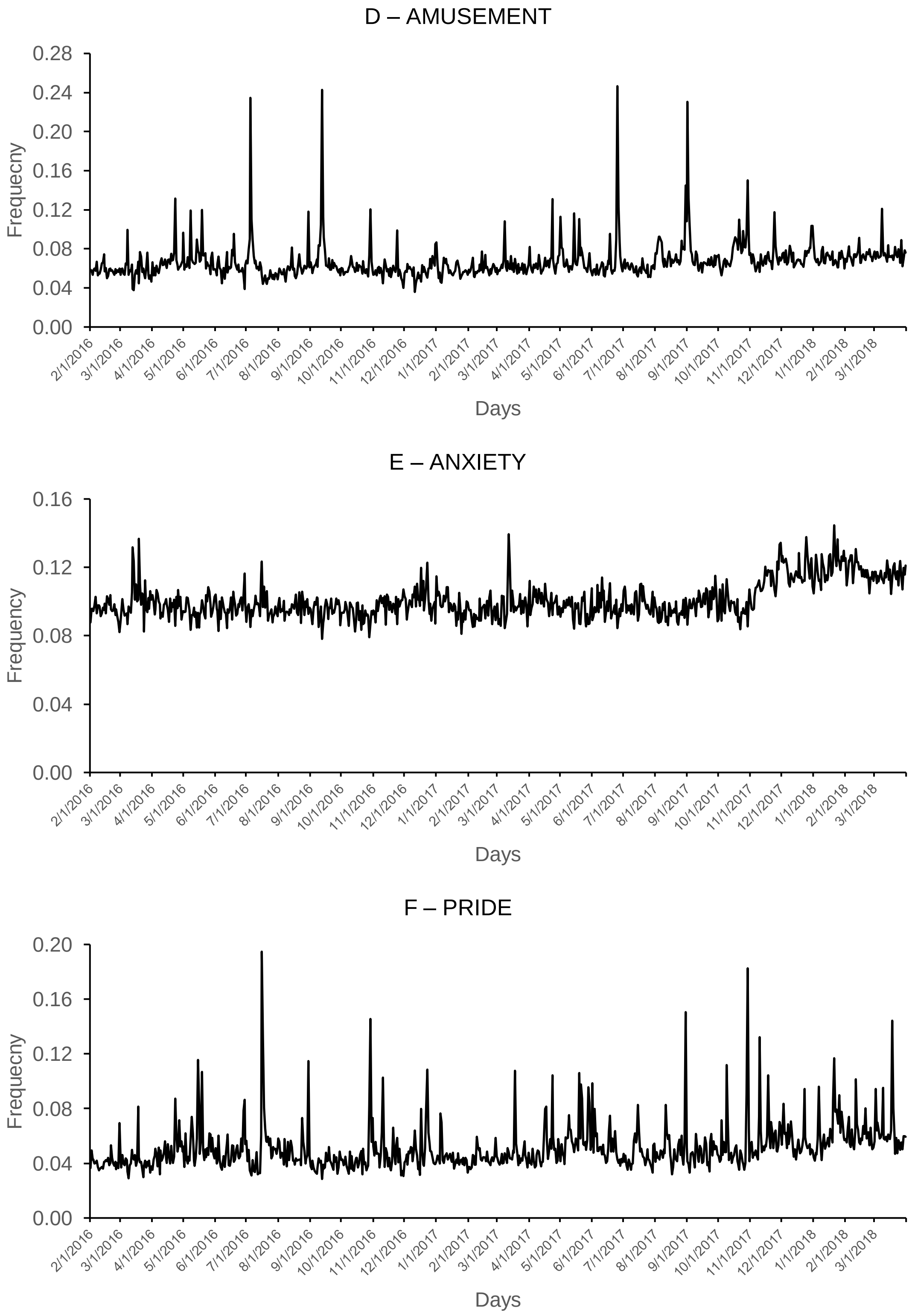

Figure S1 (cont'd). Time series plots of daily frequencies of emotions.

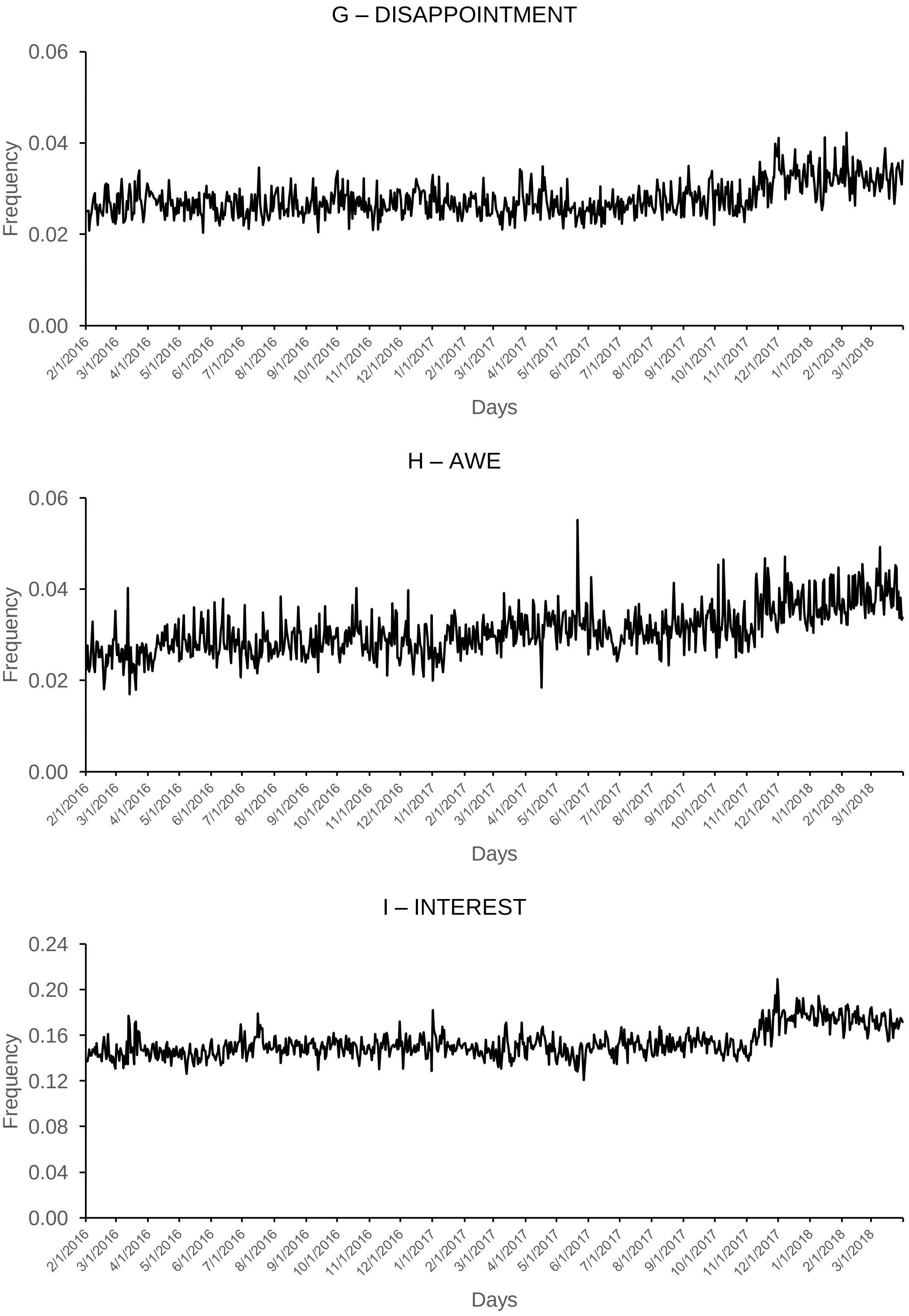

Figure S1 (cont'd). Time series plots of daily frequencies of emotions.

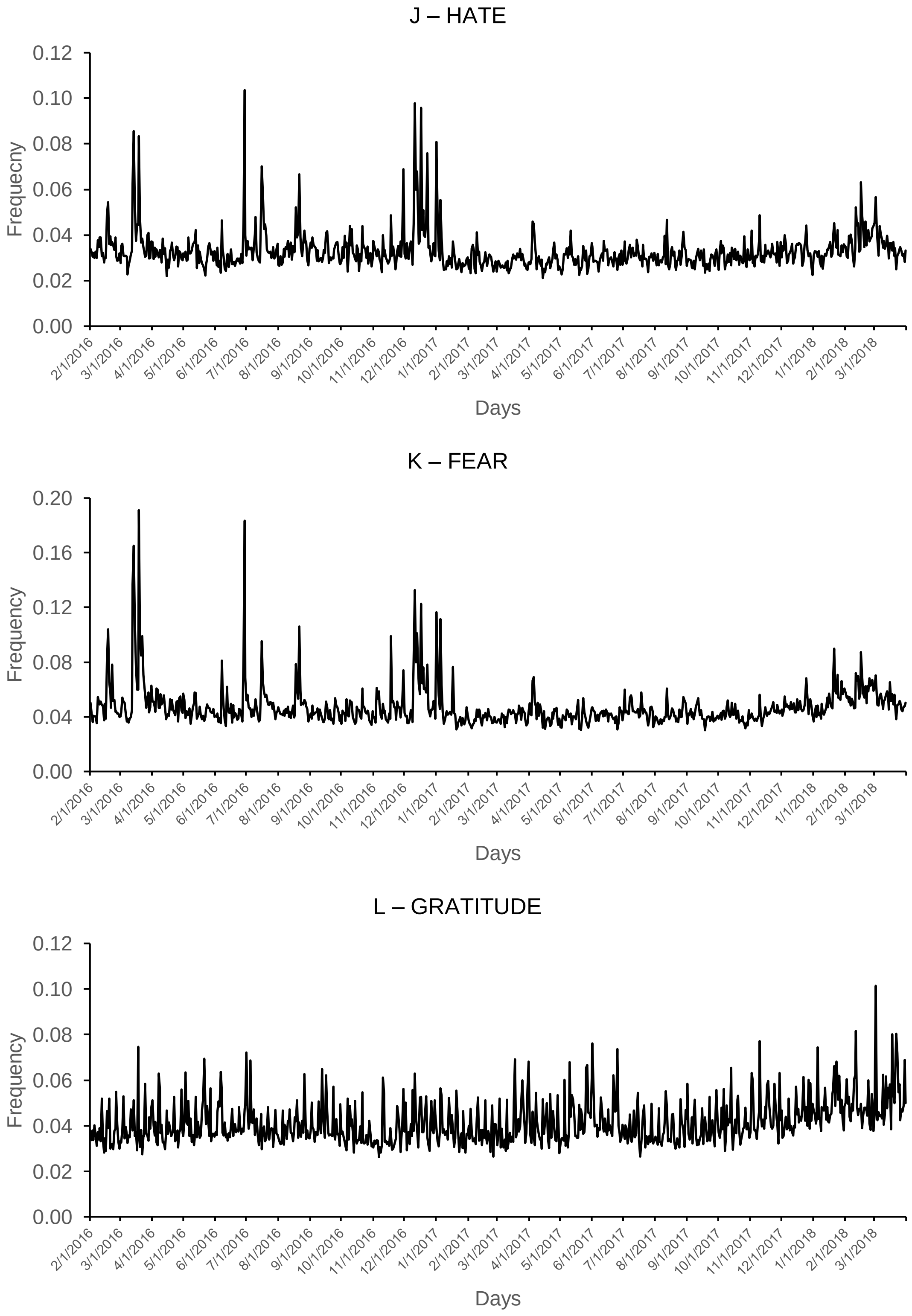

Figure S1 (cont'd). Time series plots of daily frequencies of emotions.

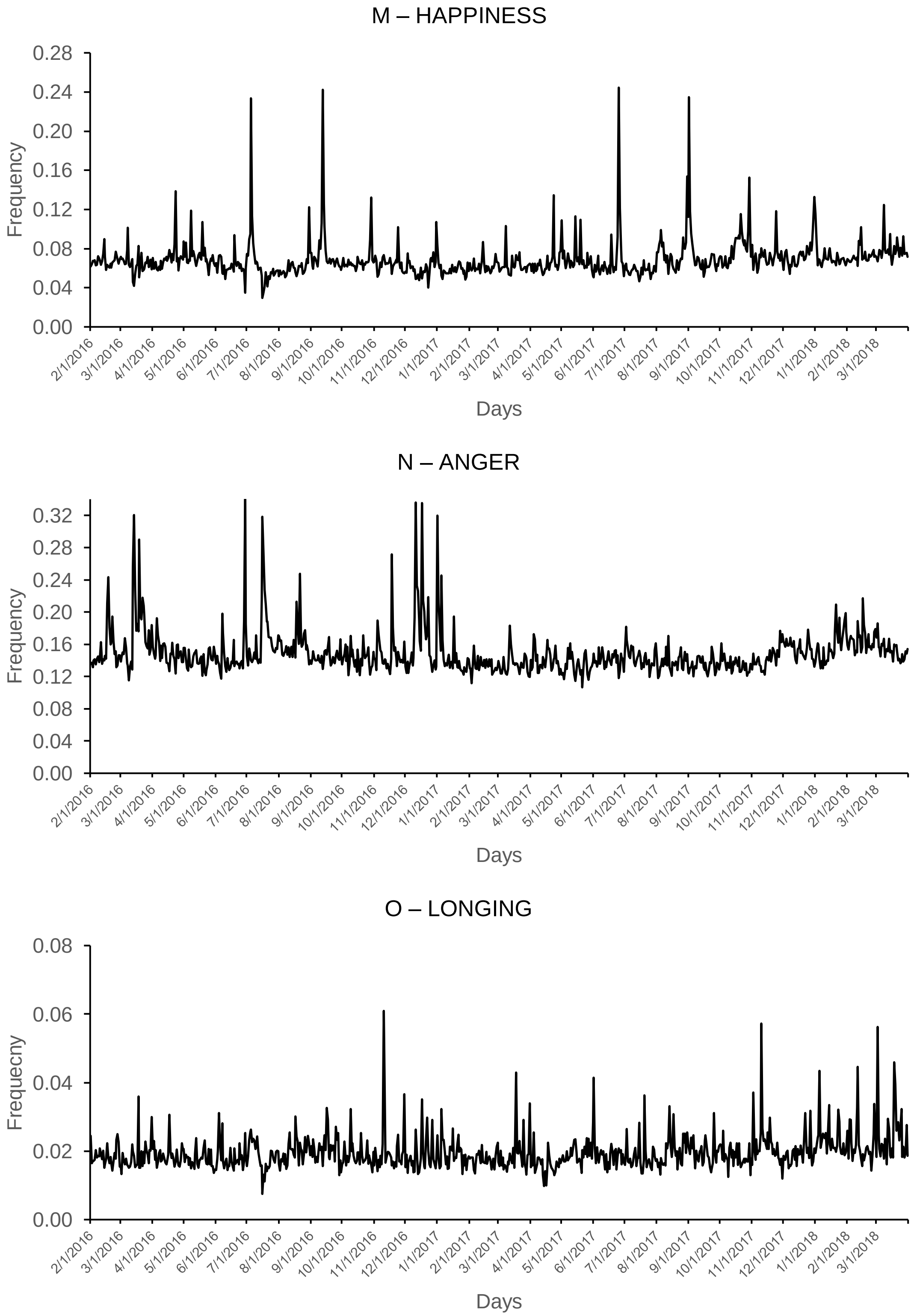

Figure S1 (cont'd). Time series plots of daily frequencies of emotions.

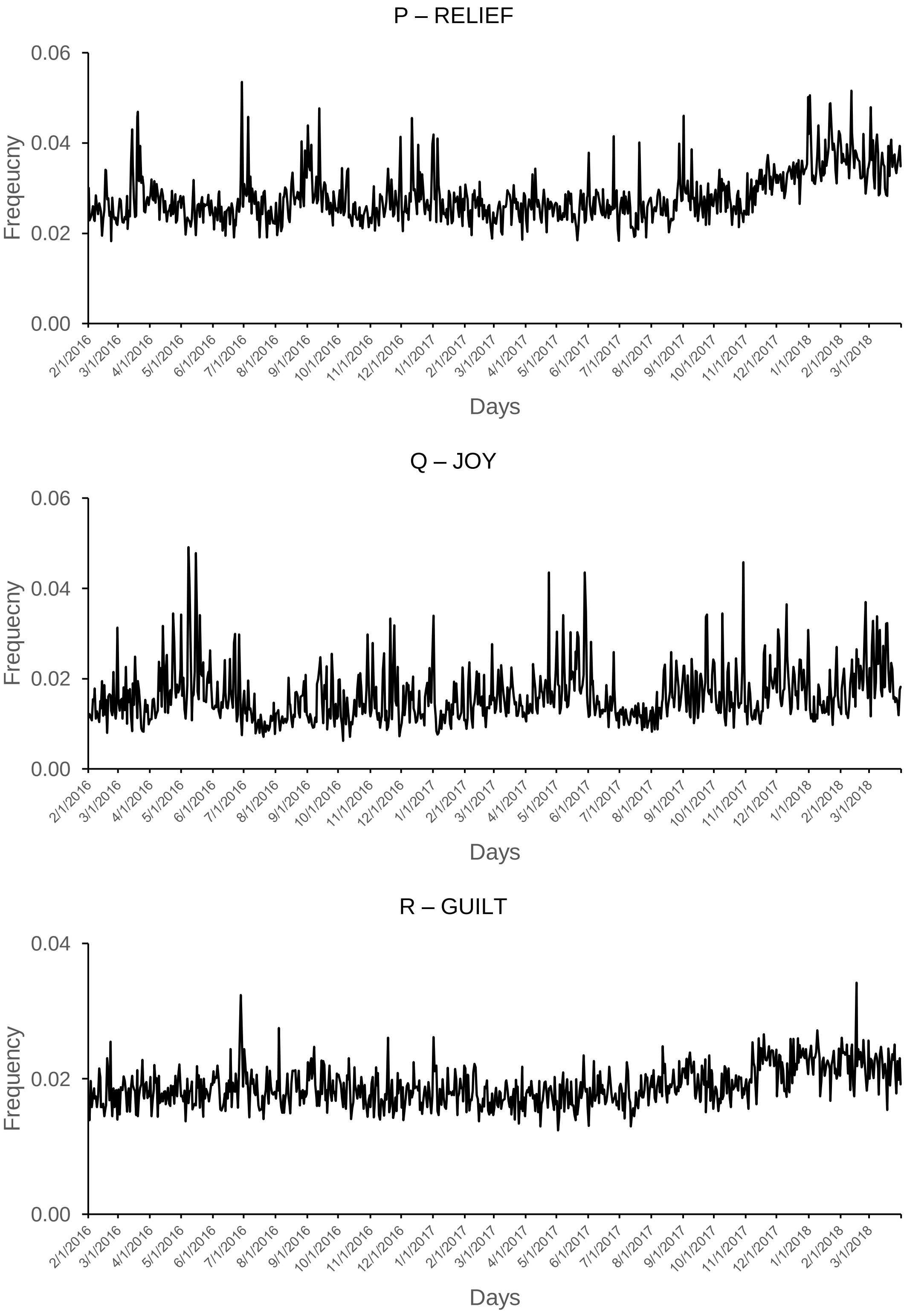

Figure S1 (cont'd). Time series plots of daily frequencies of emotions.

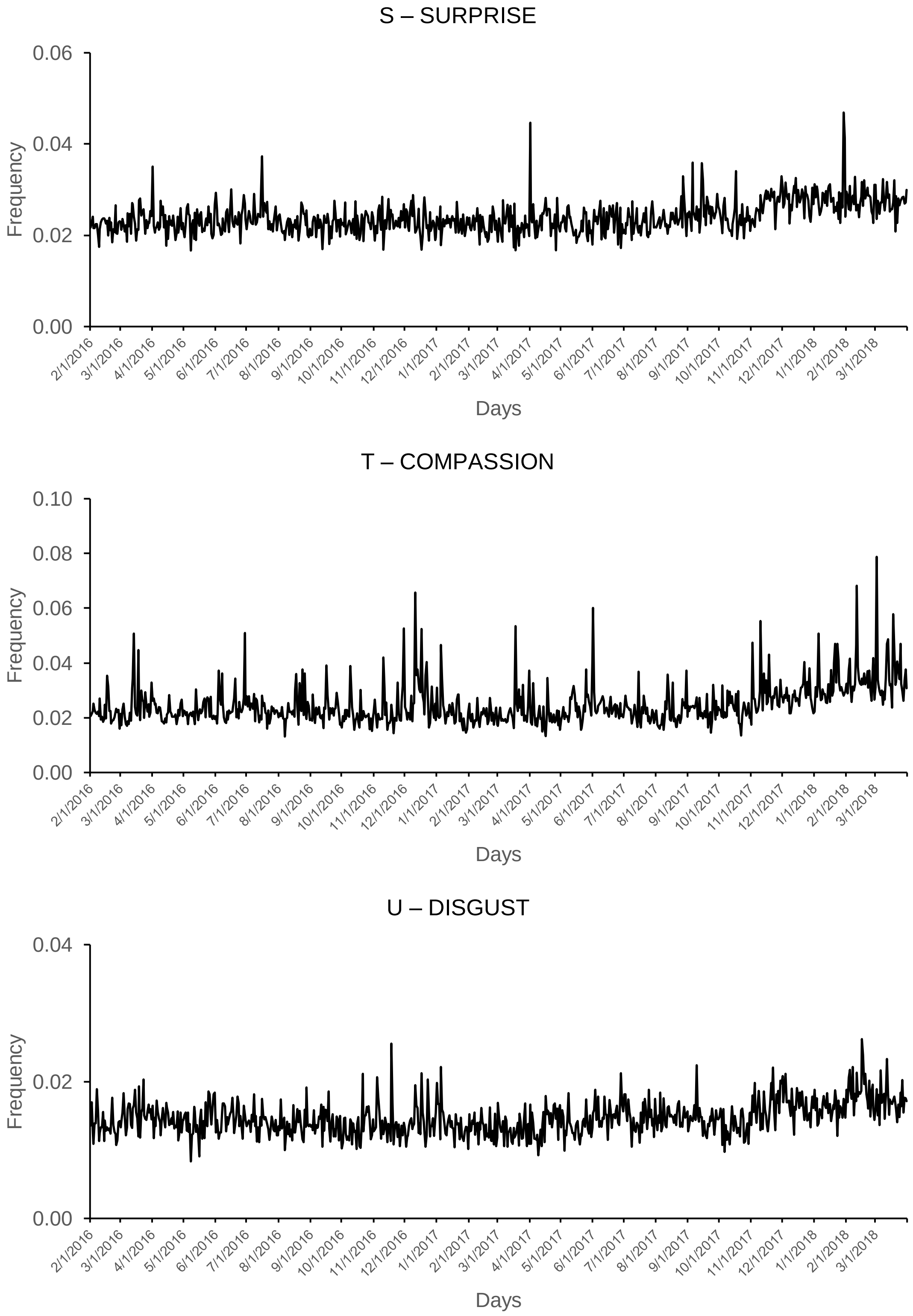

Figure S1 (cont'd). Time series plots of daily frequencies of emotions.

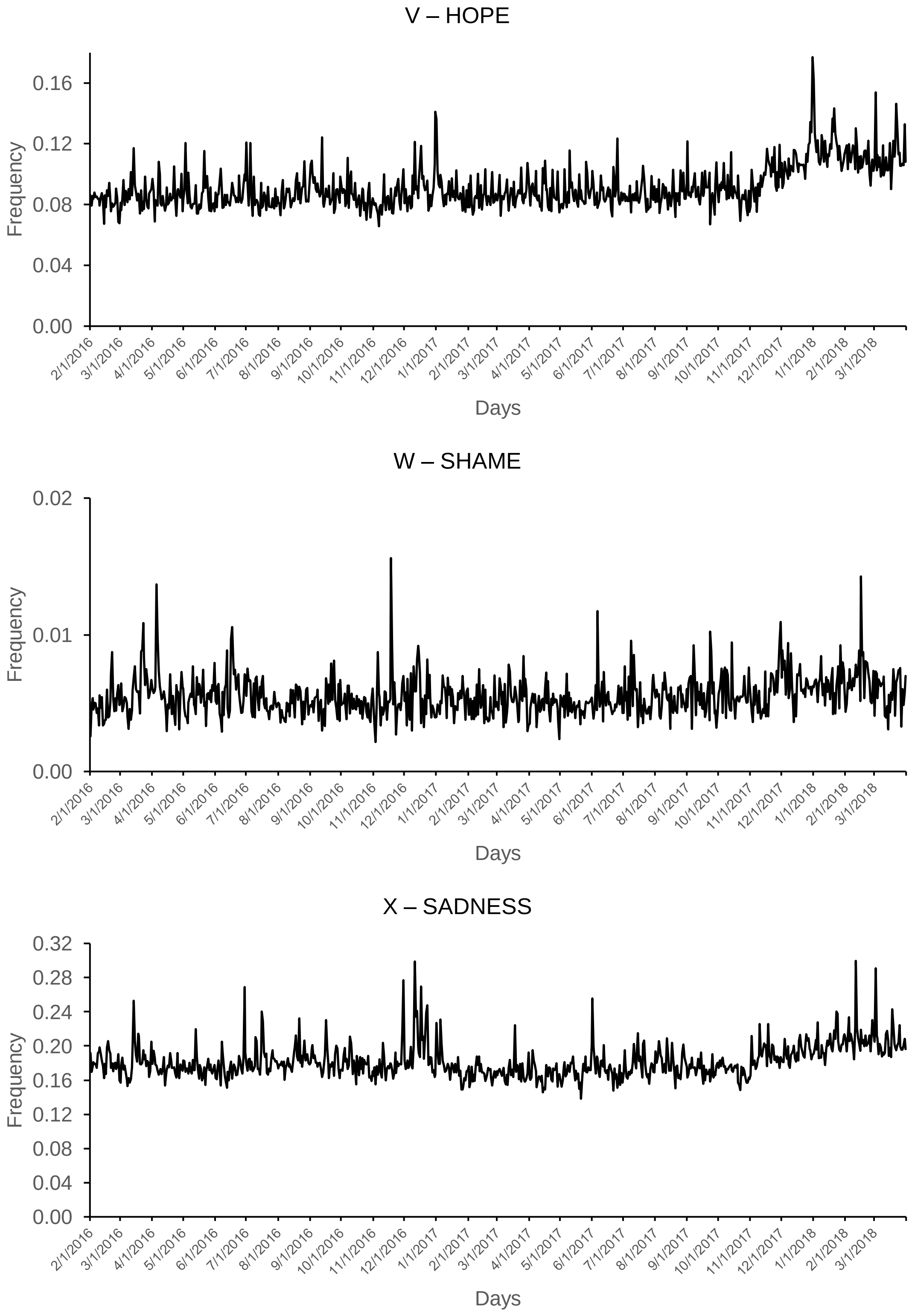

Figure S1 (cont'd). Time series plots of daily frequencies of emotions.

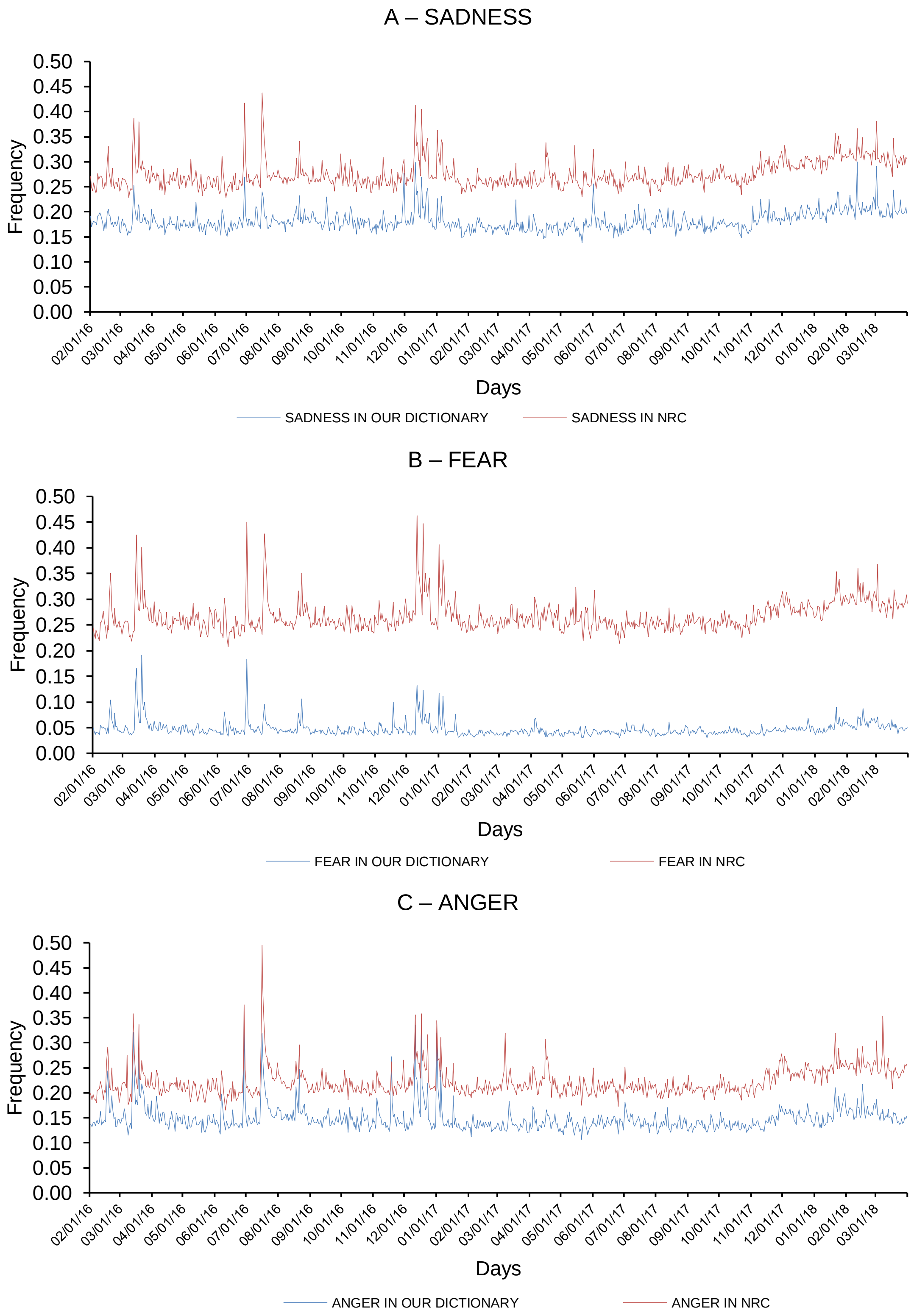

Figure S2. Comparison of NRC and our dictionary. The graphs show the comparison of daily frequencies for emotions sadness (A), fear (B), anger (C), surprise (D), joy (E), and disgust (F). Blue lines represent the daily emotional frequencies calculated using the constructed dictionaries, orange lines represent the daily emotional frequencies calculated using NRC dictionaries.

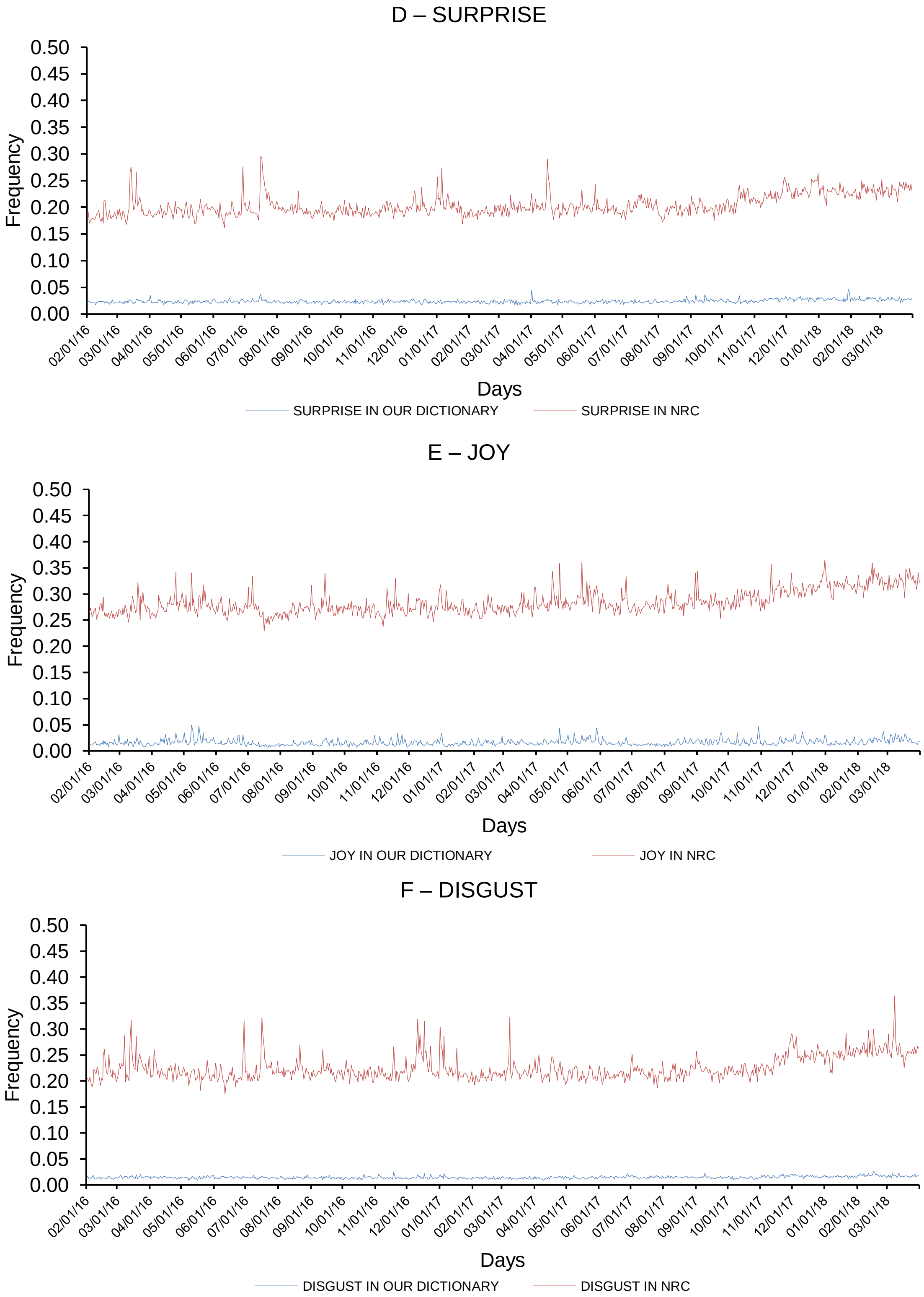

Figure S2 (cont'd). Comparison of NRC and our dictionary. The graphs show the comparison of daily frequencies for emotions sadness (A), fear (B), anger (C), surprise (D), joy (E), and disgust (F). Blue lines represent the daily emotional frequencies calculated using the constructed dictionaries, orange lines represent the daily emotional frequencies calculated using NRC dictionaries.

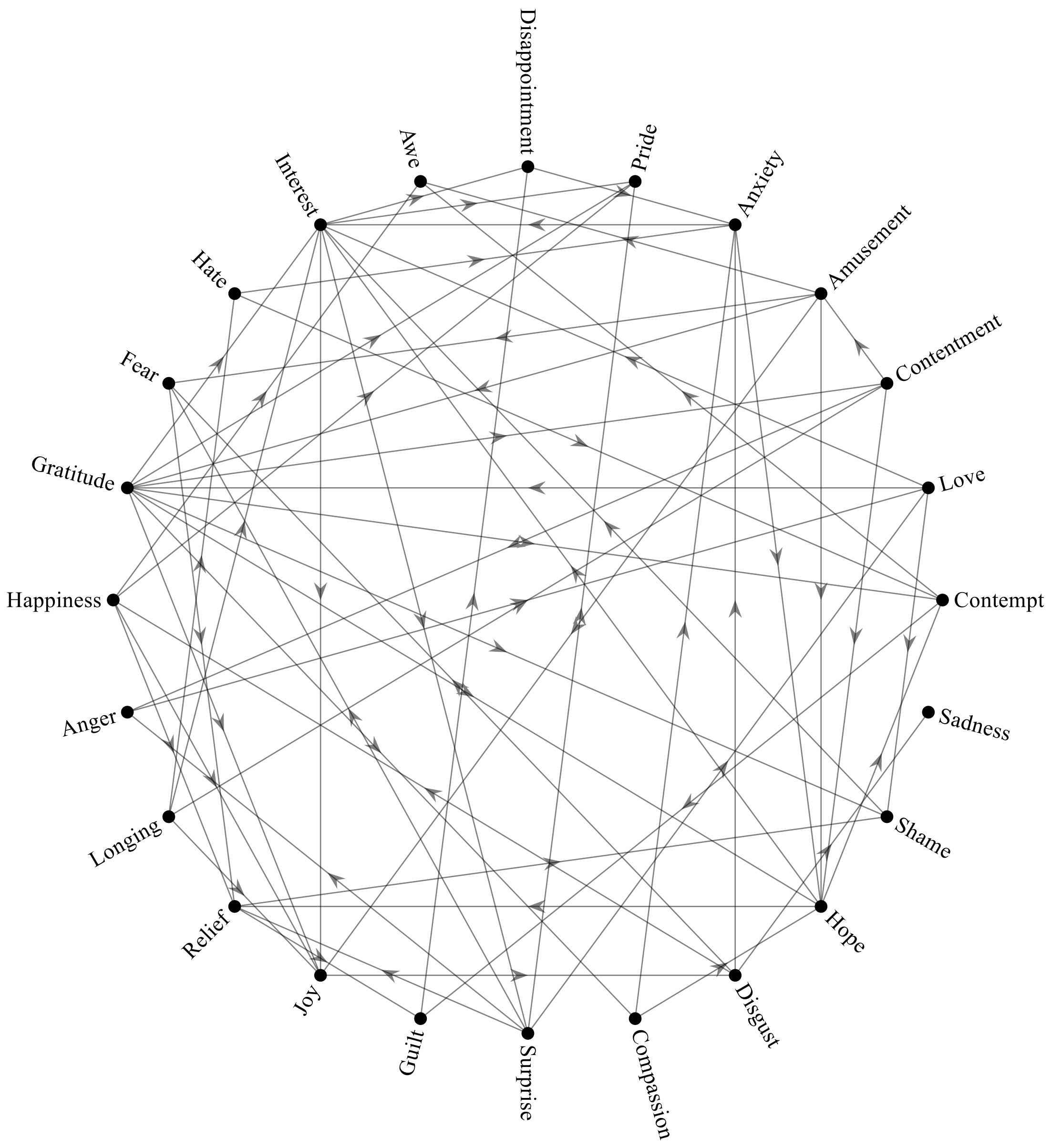

Figure S3. Network graph based on Granger Causality test results as binary outcomes. Each emotion is represented as nodes and the significant relationships (α < 0.05) between the emotions driven from the Granger Causality tests are represented by the edges.

SI References

Cowen, A. S., & Keltner, D. (2017). Self-report captures 27 distinct categories of emotion bridged by continuous gradients. *Proceedings of the National Academy of Sciences*, *114*(38), E7900–E7909.

Fontaine, J. R., Scherer, K. R., Roesch, E. B., & Ellsworth, P. C. (2007). The world of emotions is not two-dimensional. *Psychological Science*, *18*(12), 1050–1057.

Frijda, N. H., Kuipers, P., & Ter Schure, E. (1989). Relations among emotion, appraisal, and emotional action readiness. *Journal of Personality and Social Psychology*, *57*(2), 212.

Granger, C. W. (1969). Investigating causal relations by econometric models and cross-spectral methods. Econometrica: journal of the Econometric Society, 424-438.

Horn, J. L. (1965). A rationale and test for the number of factors in factor analysis. *Psychometrika*, *30*(2), 179–185.

Kapucu, A., Kılıç, A., Özkılıç, Y., & Sarıbaz, B. (2021). Turkish emotional word norms for arousal, valence, and discrete emotion categories. *Psychological Reports*, *124*(1), 188–209.

Keeling, K. B. (2000). A regression equation for determining the dimensionality of data. *Multivariate Behavioral Research*, *35*(4), 457–468.

McNair, D. M., Lorr, M., & Droppleman, L. F. (1992). *EdITS Manual for the Profile of Mood States (POMS).* Educational and industrial testing service.

Mehrabian, A., & Russell, J. A. (1974). *An approach to environmental psychology.* the MIT Press.

Mohammad, S. M., & Turney, P. D. (2013). Crowdsourcing a word–emotion association lexicon. *Computational Intelligence*, *29*(3), 436–465.

Muthén, B. O. (1994). Multilevel covariance structure analysis. *Sociological Methods & Research*, *22*(3), 376–398.

Pennebaker, J. W., Boyd, R. L., Jordan, K., & Blackburn, K. (2015). *The development and psychometric properties of LIWC2015*.

Reise, S. P., Ventura, J., Nuechterlein, K. H., & Kim, K. H. (2005). An illustration of multilevel factor analysis. *Journal of Personality Assessment*, *84*(2), 126–136.

Russell, J. A. (1980). A circumplex model of affect. *Journal of Personality and Social Psychology*, *39*(6), 1161.

Scherer, K. R. (2005). What are emotions? And how can they be measured? *Social Science Information*, *44*(4), 695–729.

Selvi, Y., Gulec, M., Aydin, A., & Besiroglu, L. (1970). Psychometric evaluation of the Turkish language version of the Profile of Mood States (POMS). *Psychiatry and Behavioral Sciences*, *1*(4), 152–152.

Smith, C. A., & Ellsworth, P. C. (1985). Patterns of cognitive appraisal in emotion. *Journal of Personality and Social Psychology*, *48*(4), 813.

Stevenson, R. A., Mikels, J. A., & James, T. W. (2007). Characterization of the affective norms for English words by discrete emotional categories. *Behavior Research Methods*, *39*(4), 1020–1024.
